# Effects of Axonal Demyelination, Inflammatory Cytokines and Divalent Cation Chelators on Thalamic HCN Channels and Oscillatory Bursting

**DOI:** 10.3390/ijms23116285

**Published:** 2022-06-03

**Authors:** Tengiz Oniani, Laura Vinnenberg, Rahul Chaudhary, Julian A. Schreiber, Kathrin Riske, Brandon Williams, Hans-Christian Pape, John A. White, Anna Junker, Guiscard Seebohm, Sven G. Meuth, Petra Hundehege, Thomas Budde, Mehrnoush Zobeiri

**Affiliations:** 1 Institute of Physiology I, Westfälische Wilhelms-Universität, Robert-Koch-Str. 27a, D-48149 Münster, Germany; tengiz.oniani@ukmuenster.de (T.O.); rahulchaudhary87@hotmail.com (R.C.); papechris@ukmuenster.de (H.-C.P.); zobeiri@uni-muenster.de (M.Z.); 2 Department of Neurology with Institute of Translational Neurology, Albert-Schweitzer-Campus 1, D-48149 Münster, Germany; laura.vinnenberg@ukmuenster.de (L.V.); petra.hundehege@ukmuenster.de (P.H.); 3 Institute of Pharmaceutical and Medicinal Chemistry, Westfälische Wilhelms-Universität, Corren-Str. 48, D-48149 Münster, Germany; j_schr46@uni-muenster.de; 4 Cellular Electrophysiology and Molecular Biology, Department of Cardiovascular Medicine, Institute for Genetics of Heart Diseases (IfGH), University Hospital Münster, Robert-Koch-Str. 45, D-48149 Münster, Germany; guiscard.seebohm@ukmuenster.de; 5 European Institute for Molecular Imaging (EIMI), Westfälische Wilhelms-Universität, Waldeyer-Str. 15, D-48149 Münster, Germany; kriske@uni-muenster.de (K.R.); anna.junker@uni-muenster.de (A.J.); 6 Center for Systems Neuroscience, Neurophotonics Center, Department of Biomedical Engineering, Boston University, 610 Commonwealth Ave., Boston, MA 02215, USA; bdwills@bu.edu (B.W.); jwhite@bu.edu (J.A.W.); 7 Neurology Clinic, University Clinic Düsseldorf, Moorenstraße 5, D-40225 Düsseldorf, Germany; meuth@uni-duesseldorf.de

**Keywords:** cuprizone model, ion channels, firing pattern, patch-clamp, computational modeling, expression system, brain slice, trace metal, thalamus

## Abstract

Multiple sclerosis (MS) is a demyelinating disease of the central nervous system that is characterized by the progressive loss of oligodendrocytes and myelin and is associated with thalamic dysfunction. Cuprizone (CPZ)-induced general demyelination in rodents is a valuable model for studying different aspects of MS pathology. CPZ feeding is associated with the altered distribution and expression of different ion channels along neuronal somata and axons. However, it is largely unknown whether the copper chelator CPZ directly influences ion channels. Therefore, we assessed the effects of different divalent cations (copper; zinc) and trace metal chelators (EDTA; Tricine; the water-soluble derivative of CPZ, BiMPi) on hyperpolarization-activated cyclic nucleotide-gated (HCN) channels that are major mediators of thalamic function and pathology. In addition, alterations of HCN channels induced by CPZ treatment and MS-related proinflammatory cytokines (IL-1β; IL-6; INF-α; INF-β) were characterized in C57Bl/6J mice. Thus, the hyperpolarization-activated inward current (I_h_) was recorded in thalamocortical (TC) neurons and heterologous expression systems (mHCN2 expressing HEK cells; hHCN4 expressing oocytes). A number of electrophysiological characteristics of I_h_ (potential of half-maximal activation (V_0.5_); current density; activation kinetics) were unchanged following the extracellular application of trace metals and divalent cation chelators to native neurons, cell cultures or oocytes. Mice were fed a diet containing 0.2% CPZ for 35 days, resulting in general demyelination in the brain. Withdrawal of CPZ from the diet resulted in rapid remyelination, the effects of which were assessed at three time points after stopping CPZ feeding (Day1, Day7, Day25). In TC neurons, I_h_ was decreased on Day1 and Day25 and revealed a transient increased availability on Day7. In addition, we challenged naive TC neurons with INF-α and IL-1β. It was found that I_h_ parameters were differentially altered by the application of the two cytokines to thalamic cells, while IL-1β increased the availability of HCN channels (depolarized V_0.5_; increased current density) and the excitability of TC neurons (depolarized resting membrane potential (RMP); increased the number of action potentials (APs); produced a larger voltage sag; promoted higher input resistance; increased the number of burst spikes; hyperpolarized the AP threshold), INF-α mediated contrary effects. The effect of cytokine modulation on thalamic bursting was further assessed in horizontal slices and a computational model of slow thalamic oscillations. Here, IL-1β and INF-α increased and reduced oscillatory bursting, respectively. We conclude that HCN channels are not directly modulated by trace metals and divalent cation chelators but are subject to modulation by different MS-related cytokines.

## 1. Introduction

The thalamus acts as both the gateway to the cerebral cortex and as a first information processing unit, with impulses arriving from motor, sensory and limbic system pathways being processed in specific thalamic relay nuclei before reaching the appropriate cortical areas [1,2]. In addition, the thalamus is a pivotal pacemaker for the generation of rhythmic oscillatory activity in the brain under physiological and pathophysiological conditions [3]. Slow synchronized oscillations in the delta and theta frequency band, which normally occur during slow-wave sleep, represent forms of thalamic dysfunction when occurring during stages of wakefulness [4,5,6]. Based on that, a number of otherwise unrelated diseases (including but not limited to absence epilepsy, central tinnitus, and schizophrenia) were commonly classified as thalamocortical (TC) dysrhythmia (TCD) syndromes. The sustained low-frequency thalamic activity was attributed to the dysfunction of certain well-defined molecular targets [7,8,9]. On the one hand, these are the low-threshold Ca^2+^ (Ca_V_3.1–3.3) channels, also termed T-type Ca^2+^ channels, generating the transient calcium current termed I_T_ [10]. Mutations of genes encoding Ca_V_ channels, especially gain-of-function mutations, have been linked to a variety of neurological and psychiatric channelopathies [11]. On the other hand, there are hyperpolarization-activated cyclic nucleotide-gated cation (HCN) channels, also termed pacemaker channels, generating the hyperpolarization-activated inward current (I_h_) [12]. Altered HCN channels function is the basis for a number of cardiac and neuronal diseases [13]. At potentials positive to about −60 mV which is normally occurring during wakefulness, TC neurons fire tonic sequences of action potentials (APs) in response to depolarizing inputs. During states of slow-wave sleep, when TC neurons are hyperpolarized to potentials below approximately −70 mV, T-type Ca^2+^ channels are de-inactivated and open in response to depolarizing inputs. Thereby, they generate a low-threshold Ca^2+^ spike (LTS) crowned by a burst of conventional APs. In TCD, such hyperpolarization is maintained during states of wakefulness. In that case, the cyclic interaction between HCN and T-type Ca^2+^ channels continues on the cellular level and the low-frequency resonant recurrent interaction between thalamic and cortical neurons persists on the network level [14]. Hyperpolarization of TC neurons may occur via knockout of HCN channels (HCN2, HCN4) or their auxiliary subunit TRIP8b, as well as reduced cyclic nucleotide-dependent modulation [7,15,16,17,18,19,20,21,22].

Multiple sclerosis (MS) is an inflammatory, demyelinating, and neurodegenerative disease of the central nervous system (CNS) that has a complex, multifactorial, polygenic basis influenced by environmental factors [23]. Grey and white matter lesions defined by focal demyelination, oligodendrocyte loss, inflammation, and neuronal damage in various brain regions characterize MS [24]. Thereby, MS pathology affects neuroaxonal and neural network structure and function [25]. Recently, alterations in connectivity centered around hubs such as the thalamus were found. Thalamic atrophy, in combination with microstructural alterations, is associated with damage of TC circuits and altered brain rhythms and may be the basis for disabled motor and cognitive functions, as well as the appearance of fatigue [24,26,27]. In association with changes in the thalamus, the cortex is relatively disconnected, and the grey matter network topology is more random than in healthy controls [25]. Recently, it was found that polymorphisms in the voltage-activated Na^+^ channels Na_V_1.8, that are ectopically expressed in cerebellar Purkinje cells substantially influence motor coordination in patients with MS via the cerebello-thalamic circuitry [28]. This finding further highlights the central role of thalamic functional connectivity and adds to the emerging evidence that changes in ion channels contribute to MS-related pathology [29].

The immune-mediated experimental autoimmune encephalomyelitis (EAE) model, the chemically-induced cuprizone (CPZ) model of general demyelination, and the focal lysolecithin model are commonly used animal models in the study of MS [30,31]. Indeed, these models have revealed alterations in brain activity and ion channel function [32,33,34,35]. In the TC system of CPZ-treated mice, the initial general axonal demyelination is followed by remyelination and is associated with a complex time course of cortical activity changes [36,37,38]. CPZ-induced demyelination was associated with strongly dampened neuronal activity in the cortex, followed by a transient phase of cortical hyperexcitability during the early stages of remyelination (7 days after CPZ withdrawal [39]). The latter may represent a damaging event that influences TC function in the long term. Demyelination is associated with the altered distribution and expression of ion channels in cortical neurons and central axons, including Na_V_1.6, K_V_1.1 and K_V_7.3 channels [32,33]. In the thalamus, focal demyelination leads to the reduced occurrence of burst firing in TC neurons, indicating an effect on ion channels involved in burst generation [38]. Indeed, CPZ-induced demyelination of epileptic C3H/HeJ mice resulted in the reduced slow rhythmic intrathalamic burst activity that was associated with a significant reduction of I_h_ in TC cells, lower surface expression of HCN channels, and the reduction in the phosphorylated form of TRIP8b (pS237-TRIP8b) [40].

Although CPZ has been used to study de- and remyelination in rodents for some time, its exact etiology remains rather elusive. Chronic CPZ treatment is mainly attributed to copper chelation and results in the cascade of events which leads to impaired activity of the copper-dependent cytochrome C oxidase (COX). This is accompanied by degenerative changes in oligodendrocytes, decreased oxidative phosphorylation, and apoptosis based on metabolic stress and widespread demyelination [41,42,43,44,45]. Since trace metal ions, such as copper and zinc, act as ion channel modulators, CPZ may alter channel function. However, it is unclear whether CPZ itself exerts direct effects. The finding that CPZ causes powerful microglial and macrophage responses points to inflammatory cytokines as potential mediators of its effects [30,46]. Following CPZ administration, microglia become proliferative and reactive, releasing type I interferons during the demyelination phase. Following CPZ withdrawal, the number of microglia rapidly reduces, which coincides with the peak of the remyelination phase [47]. At this point, these cells produce another set of cytokines, including IL-1β. Furthermore, CPZ application is associated with the production of IL-6 by astrocytes [48]. Therefore, glia-released modulators may contribute to the pathogenesis of the CNS in demyelinating diseases. Indeed, it has been shown that IL-1β and IL-6 influence passive and active membrane properties of TC neurons in C57BL/6J mice [49]. Furthermore, IFN-α and IL-1β differentially modulate I_h_ in TC neurons in epileptic C3H/HeJ mice [40].

Until now, not much has been known about the divalent cation-dependent modulation of HCN channels. While intracellular magnesium has been suggested to be a voltage-dependent pore blocker of HCN channels [50], no zinc- or copper-dependent modulation of neuronal HCN channels has yet been described. Nevertheless, data obtained from osteoclast precursor-like (RAW) cells point to zinc-induced effects on HCN channel function [51,52]. In voltage-clamp recordings, I_h_ amplitudes were increased by the application of zinc (100 µM). Interestingly, the most strongly expressed isoform in RAW cells was HCN4, the thalamic HCN channel subtype [17,53].

While feeding C57BL/6J mice with the copper chelator CPZ is assumed to target mature oligodendrocytes, the exact mechanism of action remains unclear. In particular, the direct effect of CPZ on ion channels has not been addressed. Therefore, we have herein assessed the influence of axonal de- and remyelination processes on intrinsic I_h_ in ventrobasal (VB) neurons of the thalamus and aimed to distinguish this effect from changes depending on direct substance binding to HCN channels and divalent cation chelation. For a more detailed understanding of the mechanisms underlying the changes in ion channel properties in the CPZ model of general demyelination, we separately analyzed the effects of axonal demyelination, proinflammatory cytokines, divalent cation chelators, and trace metals on I_h_ properties. Therefore, we recorded I_h_ in expression systems (HEK293 expressing mHCN2 channels; oocytes expressing hHCN4 channels) and in native thalamic neurons under control conditions and on different days after stopping CPZ feeding (Day1, full demyelination; Day7, early remyelination; Day25, complete remyelination). The effects of relevant physiological concentrations of trace metals (copper; zinc), established trace metal chelators (EDTA; Tricine; CPZ and its water-soluble derivative termed BiMPi) [54,55], and cytokines (IFN-α; IFN-β; IL-6; IL-1β) were assessed. Furthermore, cytokine effects on firing pattern of TC neurons and rhythmic intra-thalamic activity were analyzed. In addition, a mathematical modeling approach determined the dependency of slow thalamic bursting on I_h_ properties. Overall, we found that trace metals and divalent cation chelation do not directly influence I_h_. On the other hand, I_h_ current density was influenced by the degree of axonal myelination and cytokines. Moreover, we found that the availability of I_h_ current positively correlated with the occurrence of slow thalamic oscillations; however, there seem to be optimal combinations of the voltage-dependent parameters.

## 2. Materials and Methods

### 2.1. Animals

To characterize the native I_h_ in TC VB neurons, we used male C57BL/6J mice (purchased from Envigo or Charles River Laboratories, Germany). The mice were housed in groups of 3 individuals at the Institute of Physiology I (Westfälische Wilhelms–Universität, Münster, Germany). The age range of mice used for patch-clamp and field potential recordings was 4–14 weeks. CPZ treatment started when mice were 4–5 weeks old. CPZ-treated mice and aged-matched controls were sacrificed for in vitro experiments on Day1 (9–10 weeks old), Day7 (10–12 weeks old) and Day25 (13–14 weeks old) after stopping CPZ treatment and switching back to a standard chow diet. Before the start of either in vitro or in vivo experiments, mice spent at least 1 week in the housing and habituation period after being purchase from the respective breeders. All experimental procedures were performed in accordance with the principles approved by local authorities (review board institution: Landesamt für Natur, Umwelt und Verbraucherschutz Nordrhein–Westfalen, LANUV NRW). Efforts were made to minimize the number and degree of discomfort experienced by the animals used in this study.

### 2.2. CPZ Treatment

C57BL/6J male mice (aged 4–5 weeks) were subjected to CPZ treatment through the feeding of a diet containing 0.2% CPZ mixed into a ground standard rodent chow for 5 weeks; during this period, the brain of animals undergo an acute demyelination [42]. After 5 weeks of CPZ treatment, the mouse diet was shifted back to normal chow for the subsequent period. Retracting CPZ from the mouse diet allowed for the spontaneous remyelination that was complete on Day25 [42]. Age-matched control mice were housed under the same conditions and for the same period without receiving CPZ-containing food. Recordings were done on Day1, Day7, and Day25 after stopping CPZ treatment or permanently receiving a standard rodent chow diet in the case of control animals. Both 0.2% CPZ diet (TD.140800) and standard (TD.00217) rodent chow were purchased commercially (Envigo RMS GmbH, Düsseldorf, Germany).

### 2.3. Preparation of Acute Brain Slices for Whole-Cell Patch-Clamp Recordings

Animals were sacrificed according to effective German legal standards without anesthesia using DecapiCones (Braintree Scientific Inc., Braintree, MA 02185, USA), and brain tissue was rapidly removed from the skull. Brain slices (250–300 µm) were prepared as coronal sections in ice-cold oxygenated slicing solution, containing (in mM): sucrose, 200; PIPES, 20; KCl, 2.5; NaH_2_PO_4_, 1.25; MgSO_4_, 10; CaCl_2_, 0.5; dextrose, 10; pH 7.35, with NaOH. Before electrophysiological recordings, slices were transferred and kept first in a chamber with artificial cerebrospinal fluid (ACSF; content in mM: NaCl, 120; KCl, 2.5; NaH_2_PO_4_, 1.25; NaHCO_3_, 22; MgSO_4_, 2; CaCl_2_, 2; glucose, 25) at 32 °C for 20 min and thereafter at room temperature (RT) until recording began. The pH of ACSF in the incubation chamber was maintained at 7.35 by continuous bubbling with carbogen (95% O_2_ and 5% CO_2_).

### 2.4. Preparation of Acute Brain Slices for Field Potential Recordings

Animals were sacrificed under isoflurane inhalation anesthesia and brain tissue was rapidly removed from the skull. Brain slices (400 µm) were prepared as horizontal sections from mice (8–10 weeks old) using an ice-cold slicing solution containing the following (in mM): sucrose, 234; glucose, 11, NaH_2_PO_4_, 24; MgSO_4_, 10; and CaCl_2_, 0.5; pH 7.35 balanced with carbogen. Horizontal slices were obtained using a microtome (Leica VT 1200s, Leica, Wetzlar, Germany) and incubated in a carbogenated ACSF (32 °C) for at least 1 h before recording.

### 2.5. Cell Culture

Human embryonic kidney (HEK) 293 cells stably expressing murine HCN2 (mHCN2) [56,57] were maintained in Dulbecco’s Modified Eagle Medium with GlutaMAX (DMEM/GlutaMAX, Thermo Fisher Scientific, Schwerte, Germany) supplemented with 10% fetal calf serum (FCS, PAN-Biotech, Aidenbach, Germany) and 0.1% Penicillin/Streptomycin (Thermo Fisher Scientific, Schwerte, Germany) under humidified conditions at 37 °C and 5% CO_2_.

### 2.6. Oocyte Expression

For oocyte expression, the previously described cDNA template hHCN4-WT/pSGEM was linearized by NheI [58], and cRNA was generated using the in vitro transcription kit mMessage mMachine T7 (Life Technologies, Darmstadt, Germany). Defolliculated oocytes stage V and VI were purchased from EcoCyte Bioscience (Dortmund, Germany) and injected, each with 50.6 nL containing 5 ng of HCN4 cRNA. After injection, oocytes were stored for 4–5 days at 18 °C in Barth solution containing the following (mmol/L): NaCl, 88; KCl, 1; CaCl_2_, 0.4; Ca(NO_3_)_2_, 0.33; MgSO_4_, 0.6; TRIS-HCl, 5; NaHCO_3_, 2.4 and (mg/L) theophylline, 80; benzylpenicillin, 63; streptomycin, 40; gentamycin, 100.

### 2.7. Drugs

Experimentally tested trace metals (zinc, CAS No. 7440–66-6; copper, CAS No. 7440–50-8) and divalent cation chelators (EDTA, CAS No. 60-00-4; Tricine, CAS No. 5705-04-1 and CPZ, CAS No. 370-81-0) were purchased from Sigma Aldrich in powdered form. The water-soluble CPZ derivative BiMPi was synthesized as detailed below [54]. Substances were prepared as 100 mM stock solutions in distilled water and added to the bath solution. The final concentrations of compounds in ACSF varied across the experiments. CPZ was prepared as 50 mM stock in DMSO and used in a final concentration of 100 µM in ND96 buffer (final DMSO concentration of 0.2%). The CPZ stock solution was freshly prepared before each two-electrode voltage clamp (TEVC) measurement. Mouse IL-1β and IL-6 were purchased from Milteny Biotech (Bergisch Gladbach, Germany), dissolved in deionized sterile-filtered water, and aliquots were stored at −20 °C. Recombinant mouse IFN-α and IFN-β was ordered from Hycultec GmbH (Beutelsbach, Germany). Final concentrations of cytokines in whole-cell and field potential recordings were IL-1β, 1.4 nM; IL-6, 1.0 nM, for both IFN-α and IFN-β, 1000 IU ml^−1^.

### 2.8. Preparation of Water-Soluble CPZ Derivative N^1^,N^2^-Bis (1-Methylpiperidin-4-Ylidene) Oxalyl Hydrazide (BiMPi) and Purity Measurements

BiMPi was synthesized and purified according to the method published by Fries [54], followed by high-performance liquid chromatography (HPLC) analysis. Briefly, Oxalylhydrazide (12.5 g, 0.1 mol, 1 eq) was dissolved in 1 L of ethanol, then N-methyl-4-piperidone (24.3 g, 0.22 mol, 2.05 eq) and a catalytic amount of 4-toluenesulfonic acid were added. The resulting mixture was refluxed for 5 h and then cooled for 30 min in an ice bath. The formed precipitate was filtered, washed with ethanol (3 × 150 mL), and dried over CaCl_2_ to make a white solid (27.0 g, 0.09 mol, 88%). In order to determine the purity of the synthesized compound, an HPLC Merck Hitachi system with a UV detector L-7400 (λ = 210 nm) was used. The mobile phase consisted of A, H_2_O/trifluoroacetic acid (TFA) 0.05% and B, acetonitrile/TFA 0.05%. The gradient elution program was as follows: (A%): 0–4 min: 90%, 4–29 min: gradient from 90% to 0%, 29–31 min: 0%, 31–31.5 min: gradient from 0% to 90%, 31.5–40 min: 90%. The flow rate was 1.00 mL/min; injection volume: 5.0 µL; Purity (HPLC): >99% (t_R_ = 1.25 min). HPLC solvents were of gradient grade quality, and ultrapure water was used. All HPLC eluents were degassed via sonication prior to use. Below are the characteristics of the synthesized compound:

C_14_H_24_N_6_O_2_ (308.20 g/mol). Melting point: 210 °C. The given melting point was assessed by the apparatus Stuart Scientific^®^ SMP 3, uncorrected. Exact mass (APCI): *m*/*z* calculated for C_14_H_25_N_6_O_2_ [M + H^+^] 309.2034, found 309.2002. Given measurements were calibrated beforehand with sodium formate clusters and acquired using MicroTof (Bruker Daltronics, Bremen). All solvents were of analytical grade quality, and demineralized water was used. Both ^1^H-NMR (600 MHz, CDCl_3_) δ (ppm) = 10.04 (s, 2H, NH), 2.58 (s, 8H, 2-CH_2_, 3-CH_2_), 2.53 (m, J = 5.3 Hz, 8H, 5-CH_2_, 6-C*H_2_*), 2.32 (s, 6H, C*H_3_*) and ^13^C-NMR (600 MHz, CDCl_3_) δ (ppm) = 162.7 (2C, *C*-4), 155.4 (2C, *C*=O), 55.6 (2C, *C*-6), 54.3 (2C, C-2), 45.6 (C2, CH_3_), 34.8 (C2, C-5), 27.2 (C2, C-3) were measured by a Unity Mercury Plus 400 spectrometer (Varian^®^, Crawley, UK), AV400 (Bruker^®^), JEOL JNM-ECA-400; δ in ppm relative to tetramethylsilane; coupling constants are given with 0.5 Hz resolution, the assignments of ^13^C and ^1^H NMR signals were supported via 2D NMR technique, FT-IR (neat) ṽ (cm^−1^) = 3224 (N-H), 1656 (C=O), 1490 (C=N), 1371 (C-N) calculated by an IR spectrophotometer FT-ATR-IR (Jasco^®^).

Thin-layer chromatography was conducted with silica gel F_254_ on aluminum plates in a saturated chamber at room temperature. The spots were visualized using UV light (254 nm) or reagents such as cerium molybdate dipping bath, with additional heating provided by a standard heat gun. Hence, the retention factor values strongly depend on the temperature, the chamber saturation, and the exact ratio of components of the eluent (highly volatile); the given retention factor values represent only approximate values. Flash column chromatography was conducted with silica gel 600 (40–63 m, Macherey-Nagel). Parentheses include the diameter of the column, the length of the column, the fraction size, the eluent, and the R_f_ value. Unless otherwise noted, moisture-sensitive reactions were conducted under dry nitrogen.

### 2.9. Whole-Cell Voltage-Clamp Recordings in Acute Brain Slices

Voltage-dependent properties of I_h_ were characterized by whole-cell voltage-clamp recordings in TC neurons of the VB in ACSF solution at 30 ± 1 °C. The bath solution contained (in mM): NaCl, 125; KCl, 2.5; NaH_2_PO_4_, 1.25; NaHCO_3_, 1.25; MgSO_4_, 2; CaCl_2_, 2; Glucose, 10; BaCl_2_, 0.5 (BaCl_2_ was added only during voltage-clamp recordings); pH 7.25 (adjusted and maintained with continuous bubbling with carbogen.

Considering that zinc precipitates in the presence of phosphate [59,60], recordings in the presence of zinc were carried out in a NaH_2_PO_4_ free solution.

Patch pipettes were pulled from borosilicate glass (GC150T-10; Clark Electromedical Instruments, Pangbourne, UK) with a resistance of 2.5–4.5 MΩ. The internal solution (pipette solution) contained the following (in mM): K-gluconate, 88; K_3_-citrate, 20; NaCl, 10; HEPES, 10; MgCl_2_, 1; CaCl_2_, 0.5; BAPTA, 3; Mg-ATP, 3; Na_2_-GTP, 0.5. The internal solution was set to a pH of 7.35 with KOH and an osmolality of 295 mOsmol/kg. All recordings were performed on the soma of TC neurons using an EPC-10 amplifier (HEKA Elektronik, Lamprecht, Germany). The access resistance was in the range of 5–25 MΩ and was monitored throughout the experiment. Cells with an access resistance of more than 25 MΩ were discarded from the experiment. A series resistance compensation of >30% was routinely applied. Voltage-clamp experiments were controlled by the software PatchMaster (HEKA Elektronik, Lamprecht, Germany) operating on an IBM-compatible personal computer. Measurements were corrected for liquid junction potential.

**Figure 1:**
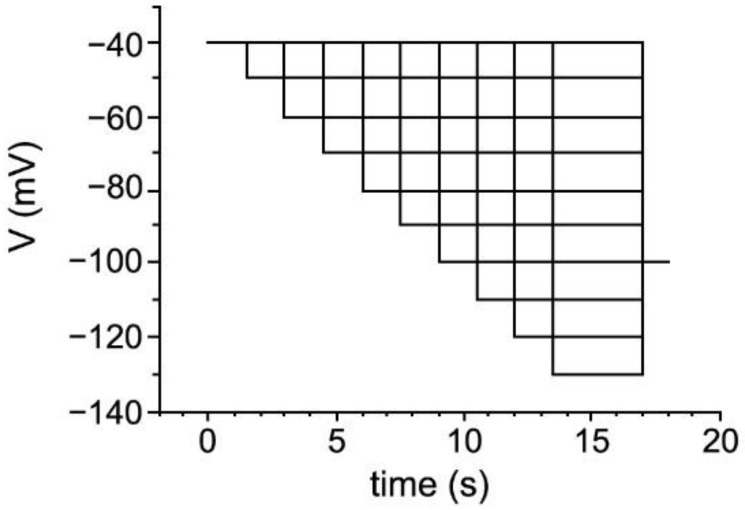
The pulse protocol for I_h_ recordings in brain slices.

I_h_ current was measured by hyperpolarizing voltage steps of −10 mV increment from a holding potential of −40 mV to −130 mV (Figure 1). The protocol was designed in order to increase the stability of whole-cell recording and to account for increasingly fast activation kinetics of the current.

The steady-state activation of I_h_, the fraction of HCN channel opening *p*(*V*), was estimated by normalizing the mean tail current amplitudes (I) 100–150 ms after stepping to a constant potential of −100 mV from variable step amplitudes (from −40 mV to −130 mV) using the following equation:

$$p\left(V\right)=\frac{I-I_{min}}{I_{max}-I_{min}\;},$$

where, *I_max_* is the tail current amplitude for the voltage step from −130 mV to −100 mV and *I_min_* represents the voltage step from −40 mV to −100 mV, respectively. I_h_ activation was fitted by a Boltzmann equation of the following form:

$$p\left(V\right)={\left(1+e^{\frac{V-V_{0.5}}{k}}\right)}^{-1}$$

where, *k* represents the slope factor. The amplitude of I_h_ was calculated by subtracting the instantaneous current amplitude from the steady-state current. The density of I_h_ (current density, CD) was calculated by dividing the maximum I_h_ current amplitude at −130 mV by the membrane capacitance obtained during whole-cell recordings. The time course of I_h_ in TC VB neurons and HEK2 cells (two components of HCN channel activation-fast and slow) was calculated by fitting the hyperpolarizing pulse step to −130 mV with a dual exponential equation:

I_h_*(t)*= *A_0_* + *A_1_e*^(−*t*/τ1)^ + *A_2_e*^(−*t*/τ2)^

where, I_h_(*t*) is the total amplitude of the current at time *t*, and *A_1_* and *A*_2_ are the respective amplitudes of the components with fast (τ1) and slow (τ2) time constants.

### 2.10. Whole-Cell Current-Clamp Recordings in Acute Brain Slices

The firing patterns and membrane properties of the TC VB neurons such as resting membrane potential (RMP), input resistance (R_in_) and the I_h_-dependent voltage sag were characterized by current–clamp recordings using a series of hyperpolarizing and depolarizing current injections (−200 pA to 500 pA) with 20 pA increments, from the RMP of cells. The length of each pulse was 1000 ms. The RMP of the TC VB neurons was measured during the step with no negative or positive current injection. The depolarizing voltage sag, an indication of I_h_ current activation, was calculated by subtracting the maximum negative voltage response after current injection to the minimum negative voltage just before the end of the current step.

### 2.11. Whole-Cell Voltage-Clamp Recordings of mHCN2 in HEK 293 Cells

HEK 293 cells expressing mHCN2 channels were seeded in 2.0 mL sterile round coverslips (MatTek Life Science, Bratislava, Slovakia) and incubated at least one night at 37 °C and 5% CO_2_. Whole-cell recordings were performed at a conventional patch–clamp setup equipped with an EPC-10 amplifier and PatchMaster software (HEKA Electronics, Lamprecht, Germany). Recording pipettes were fabricated from borosilicate glass (G150TF-10, Clark Electromedical Instruments, Pangbourne, UK). The typical electrode and series resistance was 4–5 MΩ and 8–15 MΩ, respectively. A series resistance compensation of ≥30% was routinely applied. Once the whole-cell configuration was received, the pipette solution and intracellular milieu equilibrated within 1 min. I_h_ currents of the cells were then measured and analyzed, as described above, for recordings in acute brain slices. Cells were recorded at RT under control conditions and in the presence of the test substance. The latter were applied close to the recorded cell with a multibarrel application pipette with a diameter of ~100 µm. Liquid junction potential correction was not performed. The following recording solutions were used: (1) Extracellular solution (in mM): 147 NaCl, 5 KCl, 10 HEPES, 1 MgCl_2_, 2 CaCl_2_, 0.5 BaCl_2_, 13 glucose, pH 7.3 with NaOH, oxygenated. (2) Intracellular solution (in mM): 147 NaCl, 10 HEPES, 10 EGTA, pH 7.3 with NaOH.

### 2.12. Extracellular Rhythmic Burst Activity Recordings of TC VB Neurons in Acute Brain Slices

Brain horizontal slices were transferred to an interface chamber and recordings were performed at 30 ± 1 °C. The bath solution consisted of (in mM): NaCl, 126; KCl, 2.5; NaHCO_3_, 26; NaH_2_PO_4_, 1.25; MgCl_2_, 1; CaCl_2_, 2; and glucose, 10. Rhythmic burst activity was induced through the stimulation of the internal capsule (IC) using a pair of 50–100 MΩ tungsten electrodes. Network activity in the VB complex was measured using a glass electrode (GC150T-10; Clark Electromedical Instruments, Pangbourne, UK) with the resistance of 0.5–2 MΩ. Burst firing was characterized by at least 3 high-frequency spikes with an intra-burst frequency interval of >100 Hz and an inter-burst interval of not less than 500 ms. Burst activity was analyzed from 50–100 ms after the stimulation of IC up to 2–3 s. All analyses were performed offline using Clampfit 10 (Molecular devices, US) and Peak v1.0 (Meuth IT Consulting, Butzbach, Germany) software.

### 2.13. TEVC Measurements in Oocytes

**Figure 2:**
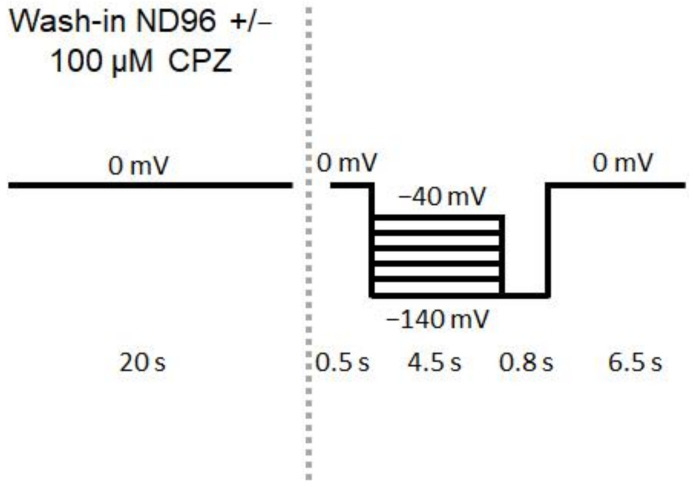
The pulse protocol for the TEVC measurements.

TEVC recordings were conducted at 22 °C using a Turbo Tec 10 CX amplifier (NPI, Tamm, Germany), a DAAD board NI USB 6221 (National Instruments, Austin, TX, USA) and the GePulse software (Dr. Michael Pusch, Genova, Italy). Recordings were performed using ND96 solution containing NaCl 96 mM, KCl 2 mM, CaCl_2_ 1.8 mM, MgCl_2_ 1 mM and HEPES 5 mM (adjusted to pH 7.4 with 1 M NaOH). CPZ test solution was freshly prepared from a 50 mM DMSO stock to final concentration of 100 µM with ND96 buffer resulting in final DMSO concentration of 0.2%. For the optimal comparability of measurements, ND96 was supplemented with 0.2% DMSO in the control recording solution. Recording pipettes were filled with 3 M KCl and had a resistance in the range of 0.5–1.5 MΩ. For stimulation of HCN4 channels, the protocol shown in Figure 2 was used. 

Before the application of different voltage steps, the ND96 + 0.2% DMSO or the 100 µM CPZ solution were washed in for 20 s at a holding potential of 0 mV. After wash-in, hyperpolarizing steps of varying voltages from −40 mV to −140 mV with the duration of 4500 ms were applied, which was then followed by an 800 ms step to −140 mV before clamping back to 0 mV holding potential (Figure 2).

### 2.14. Computational Modeling

Simulations were conducted within the NEURON simulation environment [61] by modifying a thalamic network model [62]. The original model can be downloaded at http://senselab.med.yale.edu/ModelDB/ and accessed using model number 3343 (accessed on 24 August 2021). The model simulates delta oscillations by connecting two spontaneously pacemaking TC cells and two thalamic reticular neurons (NRT) via AMPA and GABA_A+B_ synapses. Four I_h_ current parameters were modified based on experimental results acquired from whole-cell patch-clamp recordings in the TC VB neurons of CPZ-treated and their age-matched control mice: the maximum I_h_ conductance ($\underline{g_{h}}$), V_0.5_, the slope of the voltage activation curve (k), and the time constant at −130 mV ($\tau_{fast}$). The percent change in maximum I_h_ conductance was found by dividing the current density measured in each cell by the average of the combined control groups. The maximum I_h_ conductance was normalized to the default conductance in the model (10 μS/cm^2^). The voltage of V_0.5_ was shifted from the default of the model (−75 mV) to match the measured value in each given cell. The shift in voltage activation curve was equally applied to the voltage-dependent time constant curve. The voltage dependent time constant curve was then scaled to match the measured time constant at −130 mV. The I_h_ parameters were equally modified in both TC cells, while parameters in NRT cells were unaltered. The maximum potassium leak conductance was set to 0.004 μS for both TC cells. All simulations were 5 s long, and the temperature was set to 36 °C.

### 2.15. Data Analysis and Statistics

For data assembling and analyses, programs Ana (Dr. Michael Pusch, Genova, Italy), Excel (Microsoft, Redmond, WA, USA), OriginPro 2020/21 (OriginLab Corporation. Northampton, MA, USA), GraphPad Prism 7.01 (GraphPad Software, San Diego, CA, USA), and IBM SPSS Statistics 28.0 (International Business Machines Corp., Armonk, NY, USA) were used. The modulation of I_h_ current by phosphate-free medium was analyzed using repeated-measures ANOVA. CPZ feeding experiments and TEVC measurement data were analyzed using two-way ANOVA and one-way ANOVA, respectively. Here, post-hoc mean comparison Tukey test was performed. For normally distributed data, e.g., wash-in of trace metals, divalent cation chelators or cytokines and NEURON modeling data (un)paired, two-tailed Student’s *t*-test was used. The relation between two parameters was examined using non-parametric, two-tailed Spearman correlation analysis. CorelDRAW X7 (Corel Corporation, Ottawa, ON, Canada) was used for figure plotting. Differences were considered statistically significant if *p* values were less than 0.05. *, **, *** indicate *p* < 0.05, *p* < 0.01, *p* < 0.001, respectively.

## 3. Results

### 3.1. Alterations of Intrinsic Properties of TC VB Neurons following CPZ Treatment on C57BL/6J Mice

**Figure 3:**
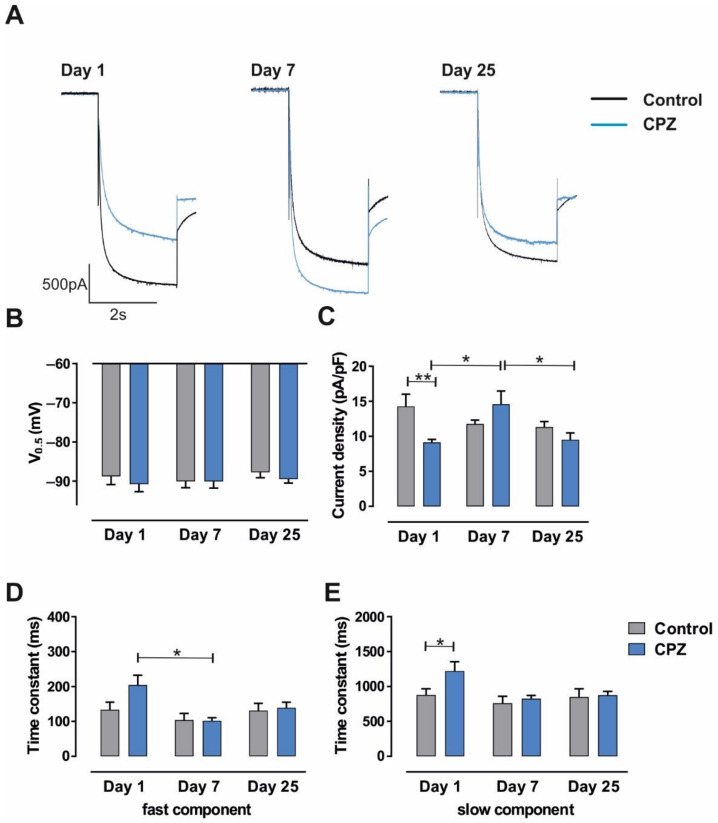
The impact of CPZ-induced de- and remyelination on I_h_ properties in TC VB neurons. (**A**) Exemplary traces of I_h_ current recorded in TC VB neurons on Day1, Day7, and Day25 of remyelination after CPZ-treatment (blue) and an age-matched control of Day1 (black). For clarity, only the hyperpolarizing step from −40 mV to −130 mV is shown. (**B**–**E**) Bar graphs displaying V_0.5_ (**B**), current density (**C**) and both fast (**D**) and slow (**E**) components of activation kinetics for CPZ-treated mice (blue) and age-matched controls (grey) for the indicated experimental days. * and **, indicate *p* values less than 0.05 and 0.01, respectively.

We assessed the influence of axonal demyelination on I_h_ current in TC neurons in a standard laboratory mouse strain. We used a toxic model of acute general demyelination in the CNS by targeting mature oligodendrocytes and feeding mice for 5 weeks with the copper chelator CPZ [37,40,63]. Different electrophysiological readouts were obtained on Day1 (full demyelination), Day7 (early remyelination) and Day25 (complete remyelination) after stopping CPZ feeding. Voltage-dependent properties of I_h_ current were assessed using whole-cell patch–clamp recordings. I_h_ current was activated by applying increasingly hyperpolarizing voltage steps (−50 to −130 mV) from a holding potential of −40 mV before stepping to −100 mV (see Figure 1). The length of the hyperpolarizing test potential was shortened with increasing hyperpolarization (3.5 s at −130 mV). Based on the expression of primarily HCN2 and HCN4 channel subunits [15,17], these neurons generate large, slowly activating inward currents following membrane hyperpolarization (Figure 3A). In order to investigate I_h_ current in isolation, inward rectifier channels were blocked by Ba^2+^ (0.5 mM), which was present in all recording solutions [19].

During the different recording conditions, evoked I_h_ currents in VB TC neurons revealed striking differences in amplitude with the highest amplitudes under control conditions and on Day7 in CPZ-treated mice (Figure 3A). Analysis of I_h_ parameters revealed that these changes were not accompanied by significant differences in voltage dependency. V_0.5_ was not different between the experimental conditions on Day1 (control: −88.75 ± 2.14 mV, *n* = 8; CPZ: −90.76 ± 1.91 mV; *n* = 6), Day7 (control: −90.03 ± 1.64 mV, *n* = 9; CPZ: −90.06 ± 1.72 mV, *n* = 6) and Day 25 (control: −87.73 ± 1.38 mV, *n* = 6; CPZ: −89.46 ± 1.02 mV, *n* = 8; Figure 3B). However, a significant reduction in current density compared to age-matched control mice was found on Day1 (control: 14.29 ± 1.74 pA/pF; *n* = 6; CPZ: 9.14 ± 0.43 pA/pF; *n* = 8; *p* < 0.01; Figure 3C). Thereafter, I_h_ current density in CPZ-treated mice significantly increased on Day7 (14.59 ± 1.88 pA/pF, *n* = 6; *p* < 0.05) and decreased again on Day25 (9.52 ± 0.98 pA/pF, *n* = 8; *p* < 0.01). No significant differences were found between control mice on different days (Day7 control: 11.78 ± 0.55 pA/pF, *n* = 8; Day25 control: 11.32 ± 0.78 pA/pF, *n* = 6).

Next, the kinetics of I_h_ current activation were analyzed (Figure 3D,E). On Day1 (control: 133.56 ± 21.72 ms, *n* = 8; CPZ: 204.08 ± 28.51 ms, *n* = 5; *p* = 0.0725), the fast time constant revealed the nominally highest value in CPZ-treated mice when compared to Day7 (control: 104 ± 19 ms, *n* = 8; CPZ: 102 ± 9 ms, *n* = 6; *p* > 0.05) and Day25 (control: 131 ± 21 ms, *n* = 6; CPZ: 139 ± 16, *n* = 8; *p* > 0.5; Figure 3D). These differences were significant (*p* < 0.05) for Day1 and Day7 in CPZ-treated mice. For the slow time constant, a significant difference was found for Day1 (control: 879 ± 89 ms, *n* = 8; CPZ: 1222 ± 134; *n* = 5; *p* < 0.05; Figure 3E). When compared to Day7 (control: 863 ± 135 ms, *n* = 8; CPZ: 825 ± 47, *n* = 6) and Day25 (control: 850 ± 118 ms, *n* = 6; CPZ: 875 ± 55 ms, *n* = 8), no further differences were detected.

In order to assess a more general measure of ion channel activity, we compared the resting membrane potential (RMP) (in the presence of Ba^2+^) of VB neurons between different recording conditions (Supplementary Figure S1). While the RMP revealed rather similar values for most conditions, a significant hyperpolarization was found for CPZ-treated mice on Day7 (control: −61.38 ± 1.38 mV, *n* = 8; CPZ: −67.67 ± 1.23 mV, *n* = 6; *p* < 0.01). When compared to Day1 (control: −61.25 ± 1.01 mV, *n* = 8; CPZ: −61.80 ± 1.87 mV, *n* = 5; *p* > 0.05) and Day25 (control: −63.17 ± 1.22 mV, *n* = 6; CPZ: −62.63 ± 2.07 mV; *n* = 8; *p* = 0.0511), a significant difference was found between CPZ-treated mice on Day1 and Day7 (*p* < 0.05).

The findings indicate that general demyelination (Day1) and early remyelination (Day7) are associated with a strong reduction and transient recovery in HCN channel activity in the thalamus, respectively. The effects on RMP further point to changes in Ba^2+^-insensitive ion channels that contribute to the RMP.

### 3.2. Modulation of VB TC Neurons in C57BL/6J Mice by Trace Metals and Divalent Cation Chelators

Since CPZ acts as a copper chelator, we assessed potential direct effects of trace metals, as well as divalent cation chelators (EDTA; Tricine; BiMPi-a water-soluble derivative of CPZ) on I_h_ current in the naïve VB TC neurons of male wild type C57B1/6J mice.

**Figure 4:**
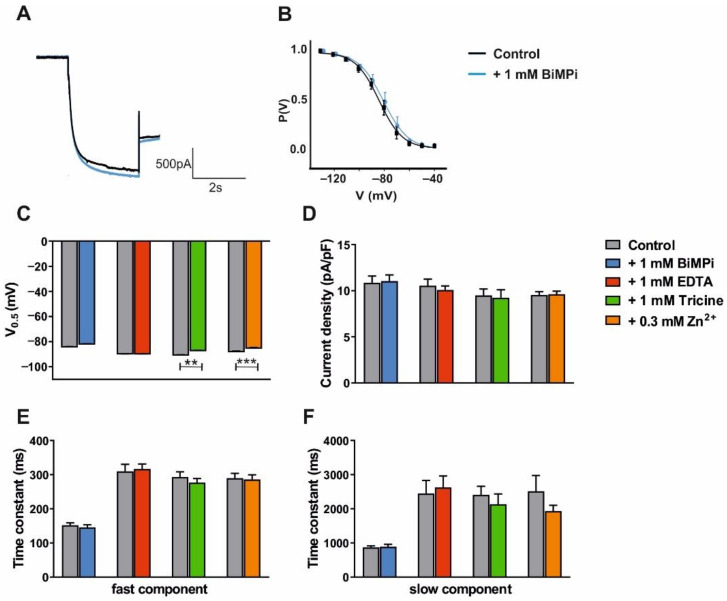
The effects of divalent cation chelators and trace metals on I_h_ current recorded in thalamic neurons. (**A**) Exemplary traces of I_h_ current recorded from VB TC neurons in control condition (black) and in the presence of 1 mM BiMPi (blue). For clarity, only the hyperpolarizing step from −40 mV to −130 mV is shown. (**B**) The mean steady-state activation curve for control and BiMPi recordings after plotting normalized tail current amplitudes against the step potential and fitting them to a Boltzmann function. (**C**–**F**) Bar graphs comparing the HCN channel V_0.5_ (**C**), I_h_ current density, the fast (**E**) and slow (**F**) component of HCN channel activation under control and experimental conditions (as indicated by color coding). ** and ***, indicate *p* values less than 0.01 and 0.001, respectively.

After acquiring control recordings, clamped cells were washed with a solution containing the test compound for 20 min before the stimulation protocol was repeated. First, the water-soluble copper chelator BiMPi (1 mM) was tested. Application of this CPZ derivate had no effect on the current amplitude (Figure 4A) and steady-state activation curve (Figure 4B). In addition, the potential of V_0.5_ (Table 1 and Figure 4C), I_h_ current density (Table 1 and Figure 4D) as well as fast (Table 1 and Figure 4E) and slow (Table 1 and Figure 4F) activation time constants were unchanged. In a similar way, the widely used divalent cation chelator EDTA (1 mM) revealed no significant effect on all analyzed parameters (Table 1 and Figure 4C–F). Next, the zwitterionic amino acid Tricine that has been widely used for zinc and copper buffering in biological systems was tested. Application of Tricine (1 mM) resulted in a significant (*p* < 0.01) depolarizing shift in V_0.5_ (Table 1 and Figure 4C). However, I_h_ current density as well as (Table 1; Figure 4D) fast (Table 1 and Figure 4E) and slow (Table 1 and Figure 4F) components of HCN channel activation were unchanged. In order to assess whether the effect of Tricine may be based on the chelation of trace metals, we next applied zinc that has been shown to exert neuro-modulatory effects on the thalamus [64]. Application of zinc (300 µM) resulted in a significant (*p* < 0.001) depolarizing shift in V_0.5_ (Table 2 and Figure 4C). However, I_h_ current density as well as the fast and slow components of HCN channel activation were unchanged (Table 2 and Figure 4D–F).

Because of the precipitating property of zinc in phosphate-bearing solutions, experiments implying zinc were performed in nominally phosphate-free ACSF [59,60]. Since the lack of phosphate may interfere with cellular energy metabolism, the I_h_ current was recorded for up to 40 min without extracellular phosphate. During this period, no significant changes occurred for all analyzed parameters (Table 2; Supplementary Figure S2).

Besides HCN channels, several other ion channels contribute to the generation of the RMP in TC neurons [65]. Therefore, we compared the RMP potential (in the presence of Ba^2+^) of VB neurons between different recording conditions. While there was a shift to more depolarized values in the presence of zinc (control: −64.29 ±1.07 mV; zinc: 57.29 ± 2.06 mV; *p* < 0.01; *n* =14) and BiMPi (control: −62.75 ± 1.81 mV; BiMPi: −59.25 ± 2.10 mV; *p* > 0.05; *n* = 8), EDTA (control: −63.67 ± 2.55 mV; EDTA: −65.33 ± 2.51 mV; *p* > 0.05; *n* = 6) and Tricine (control: −66.57 ± 1.13 mV; Tricine: −65.43 ± 1.43 mV; *p* > 0.05; *n* = 7) showed no effects (Supplementary Figure S3).

These data indicate that BiMPi and EDTA have no effect on I_h_ current parameters. The trace metal chelator Tricine has a subtle effect on the voltage-dependency of I_h_ that is concordant with the effect of applying high concentrations of zinc, and therefore it is probably not due to the chelation of ambient zinc at HCN channels. In addition, zinc exerts a depolarizing effect on the RMP.

### 3.3. Modulation of HCN2 and HCN4 Channel Subunits in HEK293 and Oocyte Cells by Divalent Cations Chelators and Trace Metals

To further characterize the effect of divalent cation chelators and trace metals on HCN channels and to address specific channel subtypes, we investigated heterologous expressed HCN2 and HCN4 channels in the following. Voltage-dependent properties of the I_h_ current were assessed using whole-cell patch–clamp recordings and applying hyperpolarizing voltage steps, as stated above.

**Figure 5:**
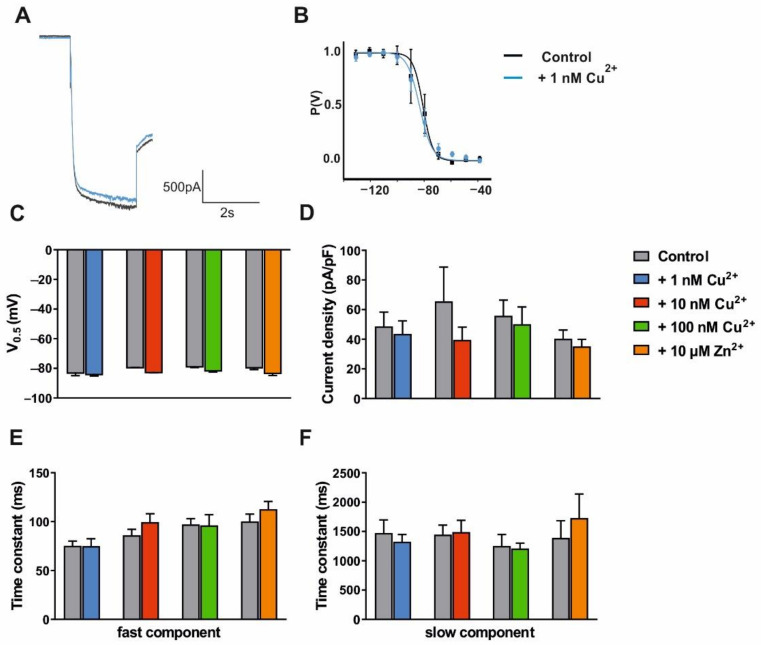
The effects of trace metals on I_h_ current recorded in HEK293 cells expressing mHCN2 channels. (**A**) Exemplary traces of I_h_ current recorded in HEK293 cells in control condition (black) and in the presence of 1 nM copper (blue). For clarity, only the hyperpolarizing step from −40 mV to −130 mV is shown. (**B**) The mean steady-state activation curve for control and copper recordings after plotting normalized tail current amplitudes against the step potential and fitting them to a Boltzmann function. (**C**–**F**) The bar graphs comparing the HCN2 channel V_0.5_ (**C**), I_h_ current density (**D**), fast (**E**) and slow (**F**) component of HCN channel activation under control and experimental conditions (as indicated by color-coding).

First, we analyzed I_h_ current in HEK293 cells stably transfected with mHCN2 channels. The application of 1 nM copper had no effect on the evoked current (Figure 5A) and the steady-state activation curve (Figure 5B). When cells were treated with different copper (1 nM; 10 nM; 100 nM) and zinc (10 µM) concentrations, no alterations in the voltage-dependence of HCN2 channels were found (Table 3 and Figure 5C–F).

**Figure 6:**
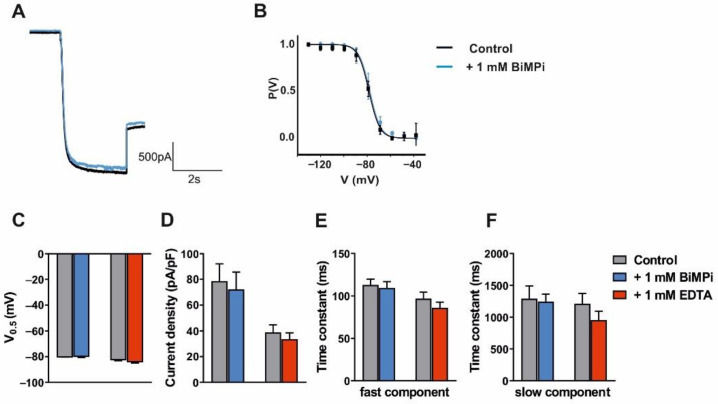
The effects of divalent cation chelators on I_h_ current recorded in HEK293 cells expressing mHCN2 channels. (**A**) Exemplary traces of I_h_ current recorded in HEK293 cells in control condition (black) and in the presence of 1 mM BiMPi (blue). For clarity, only the hyperpolarizing step from −40 mV to −130 mV is shown. (**B**) The mean steady-state activation curve for control and BiMPi recordings after plotting normalized tail current amplitudes against the step potential and fitting them to a Boltzmann function. (**C**–**F**) The bar graphs comparing the HCN2 channel V_0.5_ (**C**), I_h_ current density (**D**), fast (**E**) and slow (**F**) component of HCN channel activation under control and experimental conditions (as indicated by color-coding).

Next, we tested possible effects of divalent cation chelators (BiMPi and EDTA at 1 mM concentration) on the I_h_ current generated by mHCN2 channels. The application of BiMPi had no effect on the evoked current (Figure 6A) and steady-state activation curve (Figure 6B). In addition, both chelators showed no significant effect on other I_h_ current parameters (Table 4 and Figure 6C–F), and the components of mHCN2 channel activation were not altered.

**Figure 7:**
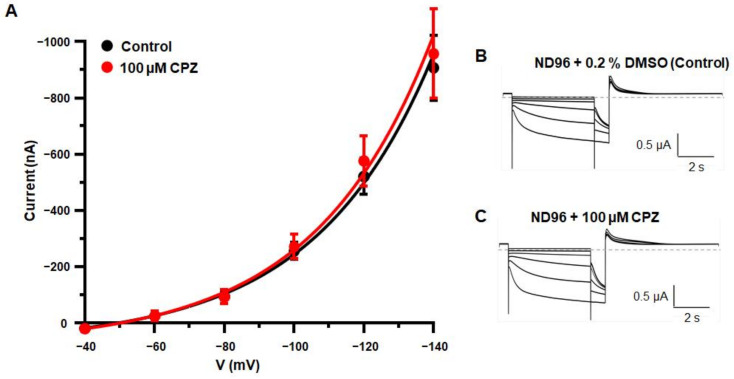
The effects of CPZ on I_h_ current recorded in oocytes expressing hHCN4 channels: (**A**) The mean currents of wildtype hHCN4 expressing oocytes in absence (black) and presence (red) of 100 µM CPZ. Values are given as mean ± SEM for independent oocytes recorded in the presence of CPZ (*n* = 8) or under control conditions (*n* = 11). (**B**,**C**) The representative current traces in the absence (**B**) and presence of CPZ (**C**).

To further determine whether I_h_ current properties are influenced by trace metal chelators, we performed TEVC measurements on hHCN4 expressing oocytes in the presence and absence of 100 µM CPZ. Different voltage pulses were applied under control condition and in the presence of CPZ from a holding potential of 0 mV, and the resulting currents were recorded (Figure 7B). Mean current amplitudes at −140 mV were −907 ± 116 nA (*n* = 11) in the absence and −957 ± 159 nA (*n* = 8) in the presence of 100 µM CPZ. The comparison of mean currents in the presence and the absence of the compound showed no significant changes in current amplitudes at any given voltage, suggesting the inactivity of CPZ at hHCN4 channels (Figure 7A).

The findings indicate that trace metals and divalent cation chelators do not directly influence I_h_ currents generated by HCN2 or HCN4 subunits.

### 3.4. Modulation of I_h_ Current, Firing Pattern and Intrinsic Properties of VB TC Neurons by Cytokines

So far, our experimental findings demonstrated clear changes in I_h_ current properties in TC neurons during different phases of remyelination following CPZ-induced demyelination. However, the application of trace metals or divalent cation chelators revealed no comparable effects on I_h_ current in brain slices and expression systems. Considering the involvement of cytokines in de- and remyelination processes [66,67] and based on their characteristic release pattern in the CPZ model [30,68], we challenged naive TC VB neurons with different cytokines (IFN-α, 1000 IU/mL; IFN-β, 1000 IU/mL; IL-1β, 1.4 nM; IL-6, 1 nM). While IFN-α and IFN-β have been suggested to be released from microglia in the demyelination phase of the CPZ model, IL-1β is released during remyelination.

**Figure 8:**
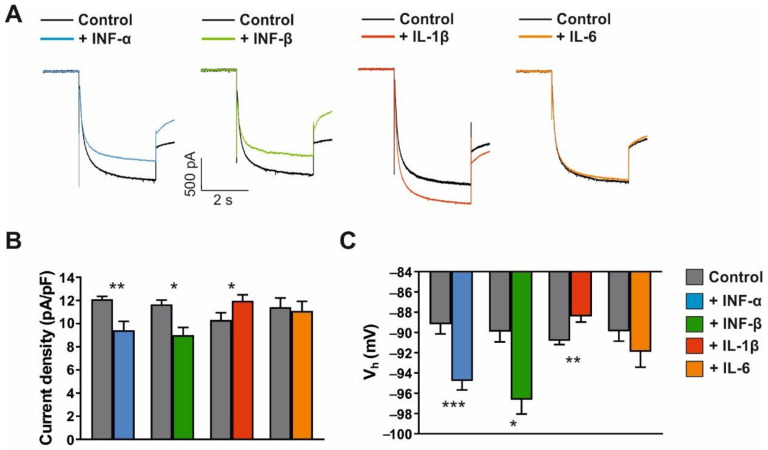
The impact of pro-inflammatory cytokines on I_h_ properties in TC VB neurons. (**A**) Exemplary traces of I_h_ current recorded in TC VB neurons under control conditions and in the presence of IFN-α, IFN-β, IL-1β and IL-6. (**B**,**C**) The bar graphs comparing the current density (**B**) and V_0.5_ (**C**) before (Control) and after application of IFN-α, IFN-β, IL-1β and IL-6. *, **, and *** indicate *p* values less than 0.05, 0.01, and 0.001, respectively.

First, cytokine effects on I_h_ current were assessed. While IFN-α and IFN-β decreased current amplitudes in TC neurons, we observed no effect of IL-6 and an increase in I_h_ amplitude induced by IL-1β (Figure 8A). These effects were associated with concordant changes in I_h_ current density (control, 11.75 ± 0.35 pA/pF vs. IFN-α, 9.53 ± 0.67 pA/pF, *n* = 7, *p* < 0.01; control, 11.66 ± 0.36 pA/pF vs. IFN-β, 9.01 ± 0.66 pA/pF, *n* = 7, *p* < 0.05; control, 10.31 ± 0.63 pA/pF vs. IL-1β, 11.96 ± 0.52 pA/pF, *n* = 7, *p* < 0.05; control, 11.41 ± 0.80 pA/pF vs. IL-6, 11.09 ± 0.83 pA/pF, *n* = 7, *p* > 0.05; Figure 8B).

The steady-state voltage-dependent activation of I_h_ current was shifted to more hyperpolarized potentials by IFN-α (control, −90.30 ± 0.82 mV vs. IFN-α, −96.67 ± 0.84 mV, *n* = 7, *p* < 0.001) and IFN-β (control, −89.75 ± 1.16 mV vs. IFN-β, −96.47 ± 1.57 mV, *n* = 7, *p* < 0.05; Figure 8C). In contrast, IL−1β shifted V_0.5_ to more depolarized potentials (control, −90.65 ± 0.53 mV vs. IL-1β, −88.24 ± 0.71 mV, *n* = 7 cells, *p* < 0.01), while IL-6 (control, −89.72 ± 1.13 mV vs. IL-6, −91.74 ± 1.67 mV, *n* = 7, *p* > 0.05) had no effect. Furthermore, the application of cytokines differentially affected the kinetics of HCN channel activation (data not shown). While the fast activation time constant was significantly decreased by IL-1β (control, 137.20 ± 4.32 ms vs. IL-1β, 127.08 ± 7.18 ms, *n* = 6; *p* < 0.05), the other cytokines had no effect. Furthermore, the slow activation time constant was decreased by IL-1β (control, 822.23 ± 24.16 ms vs. IL-1β, 724.32 ± 40.05 ms, *n* = 6, *p* < 0.05) and increased by IFN-α (control, 771.43 ± 23.95 ms vs. IFN-α, 932.70 ± 28.97 ms, *n* = 6, *p* < 0.01), IFN-β (control, 841.12 ± 24.44 ms vs. IFN-β, 933.95 ± 26.27 ms, *n* = 6, *p* < 0.05), and IL-6 (control, 795.48 ± 27.06 ms vs. IL-6, 850.72 ± 35.64 ms, *n* = 6, *p* < 0.05).

Next, the effects of cytokines on the passive and active membrane properties, as well as the firing pattern of TC neurons, were assessed under current–clamp conditions. The application of IFN-α resulted in a hyperpolarizing shift in RMP (control: −70.83 ± 1.51 mV; IFN-α: −73.50 ± 1.38 mV; *p* < 0.001; *n* = 7) and induced an increase in R_in_ (control: 165.83 ± 6.87 MΩ; IFN-α: 214 ± 13.84 MΩ, *p* < 0.01; *n* = 6; data not shown). In contrast, the application of IL-1β shifted the RMP to more depolarized potentials (control: −70.5 ± 0.43 mV; IL-1β: −68.33 ± 0.49 mV; *p* < 0.001, *n* = 6) and decreased the R_in_ (control: 170.00 ± 16.24 MΩ; IL-1β: 128.66 ± 12.74 MΩ, *p* < 0.001; *n* = 6; data not shown).

**Figure 9:**
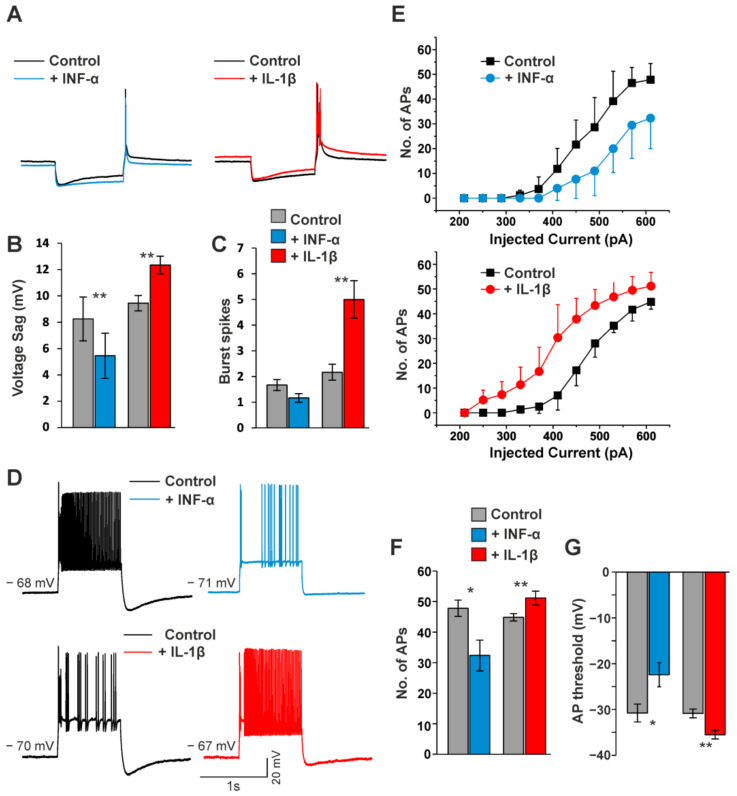
The effects of cytokines on firing properties of TC neurons of C57BL/6J mice. (**A**) Exemplary traces recorded in TC VB neurons as response to a hyperpolarizing pulse (−390 pA) under control conditions (black) and in presence of INFα (blue) and IL-1β (red). (**B**,**C**) The bar graphs showing the I_h_-dependent voltage sag (**B**) and the number of action potentials in a LTS (**C**) following release from hyperpolarization. (**D**) Exemplary traces recorded in TC VB neurons as a response to a depolarizing pulse (+450 pA) under control conditions (black) and in presence of INFα (blue) and IL-1β (red). (**E**) The graphs displaying the relationship of the injected current and the number of action potentials (APs). (**F**,**G**) The bar graphs indicating the number of APs (**F**) and the AP threshold (**G**). * and ** indicate *p* values less than 0.05 and 0.01, respectively.

The characterization of cytokine effects on the firing pattern and I_h_-dependent membrane properties in TC neurons was performed by applying hyper- and depolarizing pulses from RMP before and after the application of IFN-α and IL-1β. Upon step hyperpolarization, IFN-α decreased the I_h_-dependent voltage sag (control: 8.25 ± 1.66 mV; IFN-α: 5.45 ± 1.71 mV, *p* < 0.01; *n* = 6; Figure 9A,B) and the number of action potentials in a LTS following release from hyperpolarization (control: 1.7 ± 0.21; IFN-α: 1.17 ± 0.17; *n* = 6; Figure 9C). Upon depolarizing current steps, IFN-α reduced tonic firing (number of action potentials; control: 47.83 ± 2.66; IFN-α: 32.33 ± 5.04; *p* < 0.05; *n* = 6; Figure 9D–F) and increased their firing threshold (control: −30.77 ± 1.96 mV; IFN-α: −22.44 ± 2.60 mV; *p* < 0.05, *n* = 6; Figure 9G). Compared to IFN-α, the application of IL-1β revealed opposing effects. Upon membrane hyperpolarization, IL-1β increased I_h_-dependent anomalous rectification (control: 9.44 ± 0.58 mV; IL-1β: 12.34 ± 0.67 mV; *p* < 0.01; Figure 9A,B) and the number of action potentials in a LTS following release from hyperpolarization (control: 2.2 ± 0.31; IL-1β: 5.00 ± 0.73; *p* < 0.01; *n* = 6; Figure 9C). Upon depolarizing current injections, IL-1β caused an increase in the number of fired action potentials (control: 44.83 ± 1.22; IL-1β: 51.16 ± 2.27; *p* < 0.01; Figure 9D–F) and decreased their firing threshold (control: −30.85 ± 0.95 mV; IL-1β: −35.53 ± 0.91 mV; *p* < 0.01; *n* = 6; Figure 9G).

These findings demonstrated that different proinflammatory cytokines act on TC neurons in a different and partially opposing manner that involves the modulation of HCN channels.

### 3.5. Influence of Cytokines on Intrathalamic Oscillatory Burst Activity

**Figure 10:**
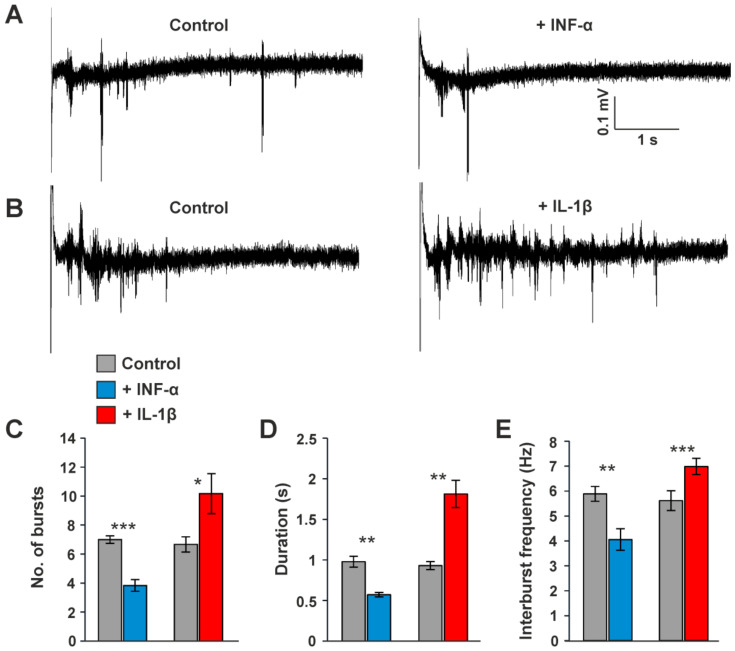
The effects of cytokines on intrathalamic burst activity in C57BL/6J mice. (**A**,**B**) Extracellular field potential recordings displaying the changes in burst activity in VB after application of IFN-α (**A**) and IL-1β (**B**) evoked by stimulating the IC. (**C**,**D**) The bar graphs showing the changes in number of bursts (**C**), their duration (**D**), and the interburst frequency (**E**) of oscillatory burst activity by inflammatory cytokines. *, **, and *** indicate p values less than 0.05, 0.01, and 0.001, respectively.

Since HCN channels exert a fundamental pacemaking function in the thalamus, we next analyzed the effect of pro-inflammatory cytokines on intrathalamic burst activity following stimulation of the internal capsule (IC; Figure 10A,B). Application of IFN-α (Figure 10A) significantly decreased the number of bursts (control: 7.00 ± 0.26; IFN-α: 3.83 ± 0.4; *n* = 7; *p* < 0.001; Figure 10C), the duration of bursts (control: 0.97 ± 0.06 s; IFN-α: 0.57 ± 0.02 s; *n* = 7; *p* < 0.01; Figure 10D), and the interburst frequency (control: 5.88 ± 0.29 Hz; IFN-α: 4.05 ± 0.43 Hz; *n* = 7; *p* < 0.01; Figure 10E). In contrast, IL-1β (Figure 10B) significantly increased the number of bursts (control: 6.66 ± 0.52; IL-1β: 10.16 ± 1.38; *n* = 7; *p* < 0.05), bursts duration (control: 0.93 ± 0.05 s; IL-1β: 1.81 ± 0.16 s; *n* = 7; *p* < 0.01) and interburst frequency (control: 5.61 ± 0.39 Hz; 6.98 ± 0.32 Hz; *n* = 7; *p* < 0.001).

These findings indicate that IFN-α and IL-1β differentially modulate intrathalamic oscillations.

### 3.6. Mathematical Modeling of Intrathalamic Network Activity

A modified intrathalamic network model, connecting two TC and two nRT neurons via AMPA and GABA_A+B_ synapses, was used to simulate delta oscillations. Based on the changes of I_h_ due to de- and remyelination, as well as the application of inflammatory cytokines (INF-α, IL-1β), the network model was changed and the influence on spontaneous rhythmic bursting was investigated.

**Figure 11:**
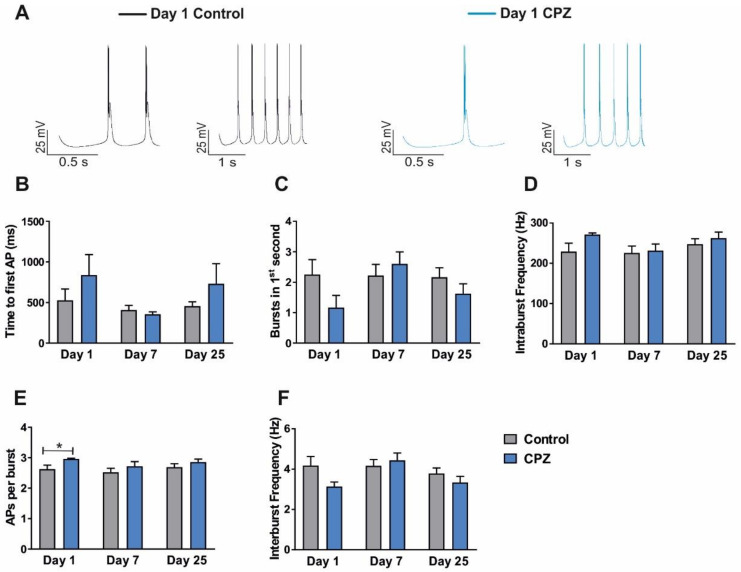
The NEURON-modeled influence of CPZ treatment on spontaneous burst activity of TC neurons. CPZ- treated mice (blue) after 1, 7, 25 days of remyelination and age-matched controls (grey) for each remyelination period. (**A**) Exemplary simulations of spontaneous burst activity of age matched controls and CPZ-treated mice on Day1 of remyelination. (**A**–**F**) The bar graphs displaying characteristics of bursting: time to first action potential (**B**), bursts within the first second of the simulation (**C**), intraburst frequency (**D**), action potentials per burst (**E**), and the interburst frequency (**F**). * indicates *p* values less than 0.05.

First, I_h_ current parameters were set to model the different phases of de- and remyelination of CPZ-treated mice and age-matched controls. Both TC neurons generated spontaneous rhythmic burst activity for all recording conditions. A comparison of burst characteristics of TC neurons on Day1 after full demyelination with age-matched controls revealed greater differences than on Day7 or Day25. The time to the first AP was increased (Day1: control, 528.5 ± 138.6 ms, *n* = 8; CPZ, 838.3 ± 252.4 ms, *n* = 6; Figure 11A,B) and the number of bursts generated in the first second of the simulation were reduced (Day1: control, 2.25 ± 0.49, *n* = 8; CPZ, 1.17 ± 0.40, *n* = 6; Figure 11C). In addition, the intraburst frequency (Day1: control, 228.8 ± 21.05 Hz, *n* = 8; CPZ, 271.2 ± 3.97 Hz, *n* = 6; Figure 11D) and the number of APs per burst (Day1: control, 2.63 ± 0.13, *n* = 8; CPZ, 2.96 ± 0.02, *n* = 6; *p* < 0.05; Figure 11E) was slightly higher under demyelinated conditions. The interburst frequency was reduced (Day1: control, 4.18 ± 0.45 Hz, *n* = 8; CPZ, 3.14 ± 0.22 Hz, *n* = 6; Figure 11F). On Day7, no obvious differences regarding the burst activity were observed compared to age-matched controls. The time to the first AP was decreased (Day7: control 408.5 ± 55.48 ms, *n* = 9; CPZ 355.1 ± 30.18 ms, *n* = 6; Figure 11A,B). The bursts generated in the first second of the simulation (Day7: control 2.22 ± 0.36, *n* = 9; CPZ 2.60 ± 0.40, *n* = 5; Figure 11C) and the intraburst frequency (Day7: control 225.5 ± 17.0 Hz, *n* = 9; CPZ 231.5 ± 16.27 Hz, *n* = 6; Figure 11D) were increased. Furthermore, the number of APs per burst (Day7: control 2.52 ± 0.13, *n* = 9; CPZ 2.172 ± 0.15, *n* = 6, Figure 11E) was slightly reduced during early remyelination, while the interburst frequency (Day7: control 4.16 ± 0.31 Hz, *n* = 9; CPZ 4.43 ± 0.37 Hz, *n* = 6, Figure 11F) increased. Parametrization of the network model with I_h_ properties representing fully remyelinated neurons after CPZ treatment demonstrated the same trend as on Day1. The time to the first AP (Day25: control 455.2 ± 53.79 ms, *n* = 6; CPZ 730.6 ± 247.1 ms, *n* = 8; Figure 11A,B) was higher under remyelinated conditions, while the number of bursts in the first second of the simulation was decreased (Day25: control 2.17 ± 0.31, *n* = 6 and Day25 CPZ 1.63 ± 0.32, *n* = 8; Figure 10C). Furthermore, the intraburst frequency (Day25: control 247.3 ± 13.59 Hz, *n* = 6; CPZ 267.4 ± 14.95 Hz, *n* = 8; Figure 11D) and the number of APs per burst (Day25: control 2.69 ± 0.12, *n* = 6; CPZ 2.85 ± 0.10, *n* = 8; Figure 11E) was slightly increased in CPZ-treated mice. The interburst frequency (Day25: control 3.78 ± 0.27 Hz, *n* = 6; CPZ 3.34 ± 0.31 Hz, *n* = 8; Figure 11F) was reduced.

**Figure 12:**
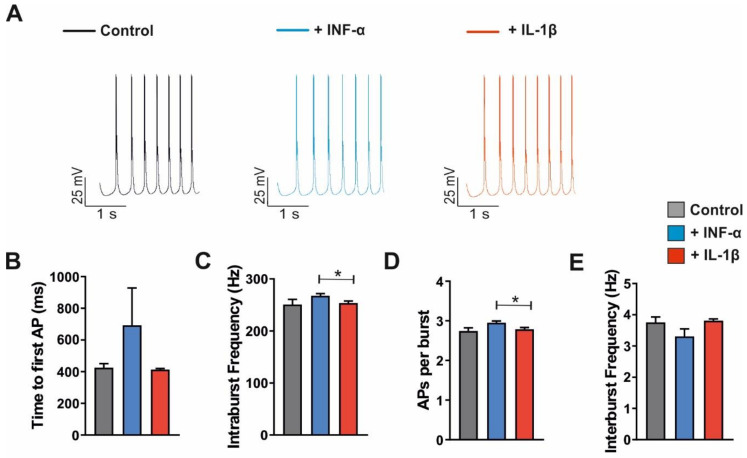
The NEURON-modeled influence of INF-α and IL-1β application on the spontaneous burst activity of TC neurons. (**A**) Exemplary simulations of spontaneous burst activity under control condition, INF-α or IL-1β application. (**B**–**E**). The bar graphs displaying characteristics of bursting: time to first action potential (**B**), intraburst frequency (**C**), action potentials per burst (**D**), and interburst frequency (**E**). * indicates p values less than 0.05.

Next, the spontaneous burst simulation model was set to I_h_ parameters observed under the application of inflammatory cytokines. Parameters obtained during INF-α application revealed delayed bursting activity compared to the control, while IL-1β did not affect the time to the first AP (control: 402.2 ± 14.9, INF-α: 692.8 ± 235.0 and IL-1β: 413.1 ± 7.04, all *n* = 6, Figure 12B). Furthermore, intraburst frequency was increased with INF- α compared to the control, while IL-1β generates no effect (control: 245.3 ± 9.61, INF-α: 267.5 ± 4.41 and IL-1β: 253.5 ± 4.15, all *n* = 6, Figure 12C). However, the difference was significant between INF-α and IL-1β conditions (*p* < 0.05). Additionally, the number of APs per burst was quantified based on the simulation which revealed a significant difference between INF-α (2.95 ± 0.05, *n* = 6) and IL-1β models (2.79 ± 0.05, *n* = 6, *p* < 0.05). Control cells generated 2.74 ± 0.09 (*n* = 7) APs per burst (Figure 12D). In the case of increased APs per burst (INF-α), the interburst frequency was slower (control: 3.89 ± 0.14, INF-α: 3.31 ± 0.24 and IL-1β: 3.81 ± 0.06, all *n* = 6, Figure 12E).

Together, these network modeling data suggest that I_h_ properties impact bursting activity in the thalamic network in silico.

**Figure 13:**
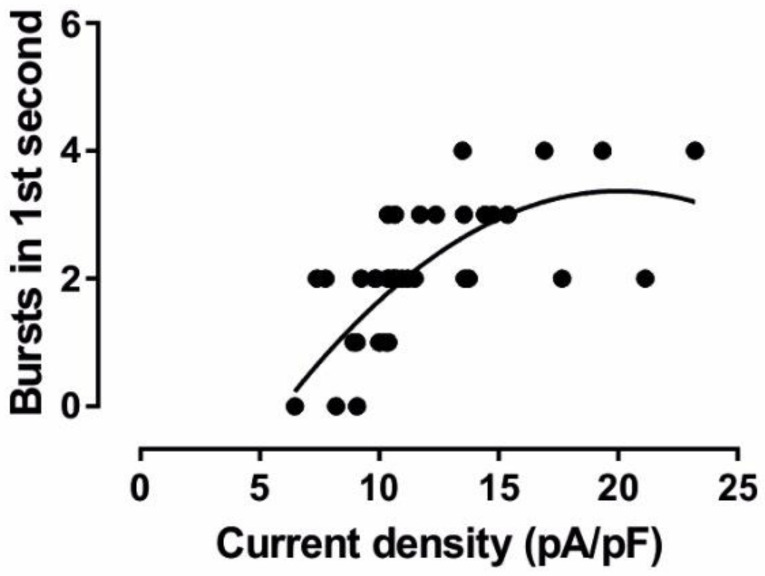
The Spearman correlation analysis with a polynomial fit of I_h_ current density (*x*-axis) obtained from voltage–clamp recordings and the number of bursts generated by the computer model within the first second (*y*-axis).

The data shown above revealed an apparent relationship between the I_h_ current density obtained from voltage–clamp recordings and the number of bursts generated by the computer model within the first second (Figure 3C and Figure 11C). Indeed, there was a clear correlation between the two parameters (Spearman correlation coefficient = 0.7313, *p* < 0.0001, *n* = 42; Figure 13) with more I_h_ current supporting more burst activity but reaching a saturating (partially decreasing) level with higher current densities.

**Figure 14:**
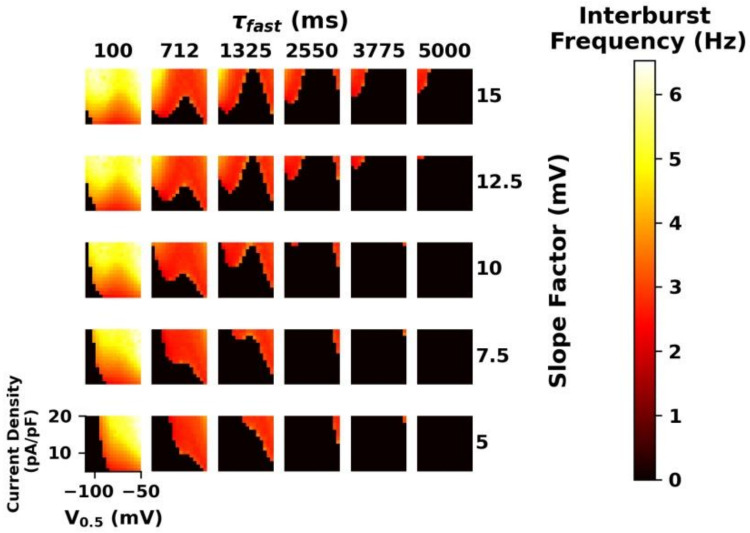
The NEURON-modeled influence of I_h_ parameters on burst activity. The computer model was parameterized with a wider parameter space of I_h_ characteristics (time constant tau fast, slope factor of the activation curve, current density and V_0.5_). The resulting oscillatory activity is shown as a density blot for different conditions. Dark colors indicate slowed burst frequency, while bright colors represent higher burst frequency.

Therefore, we next parameterized the computer model with a wider parameter space of I_h_ characteristics and analyzed the resulting oscillatory activity (Figure 14). Modulation of the time constant at −130 mV ($\tau_{fast}$) reveals a clear trend: fast opening kinetics were associated with higher burst frequencies, while slow opening kinetics entailed slower burst frequencies. The slope of the voltage activation curve was varied. In general, a less steep slope allowed for the generation of rhythmic bursts for more combinations of parameters. Furthermore, I_h_ current density and V_0.5_ exerted a strong influence on the burst frequency. The current density shows the greatest impact in the case of fast opening kinetics of (100 ms). Here, current densities larger than ~15 pA/pF led to fast interburst frequencies exceeding 6 Hz. In combination with the other parameters, alterations in V_0.5_ revealed a complex influence on bursting frequency. As the slope factor increased, the relationship between V_0.5_ and burst frequency became non-linear. At high slope factors, as V_0.5_ increased, the burst frequency switched between increasing and decreasing. This relationship was exaggerated with slower kinetics where previously slow burst activity resulted in no spontaneous bursting.

In summary, these simulations highlight the complex and highly sensitive interaction between analyzed I_h_ parameters on rhythmic thalamic bursting. Indeed, the change of only one of the identified parameters may lead to strongly changed burst activity and altered network function.

## 4. Discussion

### 4.1. Alterations in HCN Channel Activity Due to CPZ Induced de-and Remyelination

Here, we assessed the influence of axonal de- and remyelination processes on intrinsic I_h_ current in TC VB neurons by feeding C57BL/6J mice with the copper chelator CPZ for five consecutive weeks [37,39,40,42,63]. While this treatment targets mature oligodendrocytes, we aimed to distinguish this effect from changes depending on direct substance binding to HCN channels and divalent cation chelation. Electrophysiological analyses pointed to the different modulatory effects of de- and remyelination processes on Day1 (full demyelination; 1st day after CPZ withdrawal from diet), Day7 (early remyelination; 7th day after CPZ withdrawal from diet), and Day25 (complete remyelination; 25th day after CPZ withdrawal from diet). Evoked I_h_ currents in VB TC neurons revealed striking differences in amplitude with the highest values found under control conditions and on Day7 in CPZ-treated mice (Figure 3A,C). Our data demonstrate that general demyelination (Day1) is associated with a strong reduction in I_h_ current (Figure 3C), accompanied by a slowing of both components of the activation kinetics (Figure 3D). The latter may further limit the availability of HCN channels in the free membrane since the channel population in unmyelinated neurons requires a longer period to reach steady-state opening at a given potential. On Day25, when myelination was fully restored, no significant differences compared to age-matched controls, either in activation kinetics or elicited current amplitudes, were found. While there was a transient period of increased activity of HCN channels on Day7 (as compared to Day1 and Day25), the voltage dependence of HCN channel activation was constant at all measured time points (Figure 3B).

In CPZ-treated C57BL/6J mice, the initial general axonal demyelination was followed by remyelination and was associated with a complex time course of cortical activity changes [37,38,39]. It was shown that after myelin loss, the spatiotemporal propagation of incoming synaptic stimuli in the primary auditory cortex was altered. The stimulation of the internal capsule in brain slices of CPZ-treated mice had increased response latencies in the auditory cortex [39]. Moreover, the neuronal hyperexcitability displayed by an increased population spike occurrence and spike amplitudes was noticed during early stages of remyelination (Day7). This hyperexcitability was temporary, as neuronal responses were much smaller on Day1 and Day25 [39]. In addition, damage of the myelin sheath and the subsequent restoration were associated with changes of neuronal firing properties and synaptic transmission in the auditory thalamocortical system [36,37]. CPZ induced demyelination decreased the amplitude of excitatory postsynaptic potentials (EPSPs) at cortical synapses in the auditory cortex. Furthermore, even though remyelination after seven days restored synaptic strength in these synapses, it was associated with an impairment of long-term potentiation [36]. These data demonstrate that demyelination changes the ability of affected neurons to process excitatory synaptic inputs and to fire at normal action potential frequencies when stimulated [36,37,39].

It is tempting to conclude that following an initial phase of strongly dampened neuronal activity, the transient period of cortical overexcitation may represent a damaging event that alters thalamocortical network function in the long term. Our findings point to the remarkable coincidence of HCN channel downregulation in fully demyelinated neurons and temporal increased availability during the ongoing remyelination process (Figure 3), suggesting the involvement of HCN channels in these activity changes, especially considering that TC rhythms are strongly regulated by HCN channels and that the action potential conduction velocity in central axons is modulated by HCN channel blockers and cyclic adenosine monophosphate (cAMP) [69].

The changes in neuronal firing patterns based on alterations of HCN channels have been demonstrated for different types of neuronal inflammation before. Intracerebroventricular injections of lipopolysaccharide (LPS) results in the reduction of HCN1 channel expression in the hippocampal CA1 pyramidal neurons (reduced by 37%) and a consequent alteration of neuronal integrative properties [70]. Using whole-cell recordings in distal dendrites of CA1 pyramidal cells at stratum radiatum (SR) and stratum lacunosum moleculare (SLM), a 50% decrease in the amplitude of the I_h_ after 24 h of LPS treatment was found. LPS-induced downregulation of HCN1 expression is also associated with alterations in channel kinetics: 24 h after LPS treatment, the time constants were increased by 50% and V_0.5_ was shifted by −7 mV [70]. These changes in HCN1 channel properties were associated with the increased temporal summation of synaptic inputs.

Notably, the HCN channel modulation via CPZ-induced de- and remyelination processes seems to depend on the investigated mouse strain. While both epileptic C3H/HeJ and non-epileptic C57BL/6J mice display lowered I_h_ currents in TC VB neurons on Day1 and properties like in control mice on Day25, there were diverging results on Day7. CPZ-treated C57BL/6J mice are characterized by increased of HCN channel activity on Day7, while C3H/HeJ mice showed decreased current amplitudes (Figure 6; [40]). Moreover, CPZ-treated C3H/HeJ mice had slowed HCN channel activation kinetics and more hyperpolarized steady-state activation curves on both Day1 and Day7 [40]. In C57BL/6J mice (Figure 3), the slower activation kinetics was only seen on Day1. A number of factors may have contributed to these differences. The I_h_ current density in thalamic TC neurons in rodent models of absence epilepsy was increased compared to non-epileptic strains and may not be further enhanced by additional experimental paradigms [9]. Furthermore, the degree of demyelination after CPZ diet seems to be influenced by strain, age, and gender of the used experimental mice [42]. Commonly, juvenile mice are more prone to demyelination induced by CPZ diet than aged mice that require much higher doses of CPZ for the same effect. This observation may be partially explained by the difference of myelin and transcription factor gene expression in differently aged mice [71]. In C57BL/6J mice, CPZ treatment leads to higher levels of demyelination in juvenile (3 weeks) and young-adult mice (6 weeks), correlating with the major reduction of myelin basic protein and the loss of mature oligodendrocytes compared to middle-aged (8 weeks) mice [72]. Gender resistance towards CPZ treatment seems to be also dependent on the strain of used mice. Swiss and SJL/J female mice show no significant demyelination compared to their male counterparts [73,74], whereas in the BALB/cJ strain males even display resistance [75]. Furthermore, there seems to be no gender difference in C57BL/6J mice [76]. While appropriate data are not available, similar considerations may apply for C3H/HeJ mice.

Nevertheless, a potential molecular mechanism involved in HCN channel regulation following CPZ treatment has been recently identified in C3H/HeJ mice. Reduced HCN channel activity in C3H/HeJ mice was associated with the downregulation of the phosphorylated form of pS237-TRIP8b [40]. The auxiliary subunit TRIP8b critically regulates the surface expression of HCN channels in TC VB neurons [18], and it has been experimentally shown that TRIP8b can affect both gating and trafficking of HCN channels across the brain [77,78]. Although in these conditions, the total protein expression of TRIP8b remains unchanged, it seems that downregulation of pS237-TRIP8b results in the similar percentile of lowered surface expression of HCN4 channels on Day1 in C3H/HeJ mice [40]. It should be noted that TRIP8b was decreased under neuroinflammatory conditions [70] and the phosphorylated form of TRIP8b displays enhanced binding to HCN channels [79], indicating that during demyelination this form might play a major role in HCN channel modulation. Thus, it may be speculated that the difference of HCN channel modulation during de- and remyelination processes in C3H/HeJ and C57BL/6J mice might be dependent on the different levels of pS237-TRIP8b, resulting in different trafficking of HCN channels in TC neurons, especially considering that demyelination is already associated with altered distribution and expression of ion channels (like Na_V_1.6, K_V_1.1 and K_V_7.3) in cortical neurons and central axons [32,33]. However, the state and level of auxiliary subunit TRIP8b or its phosphorylated forms expression in C57BL/6J mice following CPZ treatment has yet to be determined.

### 4.2. HCN Channels Are Not Directly Modulated by Trace Metals and Divalent Cation Chelators

Although CPZ has been used to study de- and remyelination in rodents for some time, its exact etiology has remained rather elusive. Upon chronic CPZ treatment, the metabolism of copper and zinc is disturbed in the mouse brain [45]. CPZ targets metalloenzymes and results in a cascade of physiological events. These include impaired activity of the copper dependent cytochrome C oxidase (COX), degenerative changes in oligodendrocytes (like induced formation of megamitochondria), and decreased oxidative phosphorylation, ultimately leading to apoptosis based on metabolic stress and widespread demyelination [41,42]. CPZ intoxication is mainly attributed to copper chelation, consecutively leading to the altered function of many different enzymes. Accordingly, the level of copper in treated animals was reduced by 50% compared to controls after CPZ treatment. In addition, reduced COX activity (23% lower reaction rate) was detected [43,44]. Since copper acts as an essential component of mitochondrial COX, reduced levels of copper in the brain induce the formation of megamitochondria in oligodendrocytes.

Besides the formation of megamitochondria in oligodendrocytes, CPZ treatment may be expected to induce changes in ion channel modulation. Divalent trace metal ions, such as zinc and copper, function as signaling molecules in the CNS and are released from synaptic terminals of distinct neuronal cell types, thereby influencing neuronal excitability and synaptic plasticity [80,81]. Both zinc and copper occupy an allosteric binding site on Ca_V_2.3 channels and interfere with voltage-dependent gating, demonstrating that Ca_V_2.3 voltage-gated calcium channels are one of the most sensitive molecular targets of trace metals [59,82,83,84]. Moreover, based on the specific channel subtype, zinc can act both as a pore opener or blocker of members of the T-type channel family (Ca_v_3.1; Ca_v_3.2; Ca_v_3.3). While a slowing of inactivation kinetics was found for Ca_V_3.1 and Ca_V_3.3, a prolongation was found for the deactivation kinetics of Ca_V_3.3 currents (∼100-fold). Consequently, the application of zinc results in an increase of Ca_V_3.3 current in action potential clamp experiments, while Ca_V_3.1 and Ca_V_3.2 currents are reduced [81,85]. Besides voltage-gated ion channels, zinc modulates ligand-gated ion channels in the thalamus. It has been shown that synaptic release of zinc modulates GABAergic responses in VB and NRT, as well as in sensorimotor cortex, and that in these regions zinc displays concentration-dependent inhibition of GABA-evoked currents [64,86]. Compared to zinc, much less is known about the action of copper in the thalamus. Studies aimed at defining the histopathological consequences of copper deficiency have demonstrated that this condition results in myelopathies and glial activation, but also neuronal degeneration that was generically described as spongiform and necrotic zones distributed in cortex and thalamus [87].

Considering the disturbed copper and zinc metabolism in the brain due to CPZ treatment [45] and based on the modulatory influence of trace metals on different ion channels [64,80,81,85,86], we here investigated whether the CPZ-induced changes in HCN channel properties were related to ion chelation or direct compound effects. Therefore, we applied physiological relevant concentrations of copper and zinc, as well as established trace metal chelators [59,84], such as EDTA, Tricine, and a water-soluble derivative of CPZ (BiMPi) to determine possible effects on I_h_ properties in native thalamic neurons as well as in HEK293 cells that were stably transfected with mHCN2 channels. For comparison, CPZ was administered to hHCN4 channels expressed in oocytes [58].

Electrophysiological readouts of high concentrations of BiMPi application in native TC VB neurons (Figure 4) or HEK293 cells (Figure 5 and Figure 6) and CPZ administration in oocytes (Figure 7) revealed no changes in HCN channel properties (voltage-dependence, channel activation kinetics, current density) and RMP (Supplementary Figure S3). Moreover, high molarity of divalent metal buffers (EDTA and Tricine) does not have a significant effect on any of the measured I_h_ current parameters. Only, the trace metal chelator Tricine showed subtle effect on the voltage-dependency of I_h_ (Figure 4), however this effect is concordant with the effect of applying high zinc concentrations and therefore probably not based on the chelation of ambient zinc after application of Tricine to TC VB neurons. Therefore, the absence of major effects of divalent metal chelators (BiMPi, CPZ, EDTA, Tricine) on HCN channel activity is in line with the finding that copper did not modulate HCN channels. However, zinc showed a minor depolarization in the RMP of the investigated cells (Supplementary Figure S3) and on the voltage-dependence of HCN channels (Figure 4). Since zinc tends to precipitate in the presence of phosphate, the application of zinc requires phosphate-free bath solutions [55,60]. However, we demonstrated that withdrawal of phosphate from the bath solution did not affect HCN channel properties or cells survivability of the investigated cells for up to 40 min (Supplementary Figure S2).

Interestingly, osteoclast precursor-like (RAW) cells exert zinc-induced effects on HCN channel function [51,52]. While the application of zinc to RAW cells led to membrane hyperpolarization, we noted a depolarizing effect in our recordings. Furthermore, in voltage–clamp recordings of RAW cells, I_h_ current amplitudes were increased by application of 100 µM zinc [51,52]. In the present study, 300 µM zinc did not have any effect on I_h_ current in TC VB neurons (Figure 4). As HCN4 channels are a major isoform in RAW cells and thalamic neurons [17], these differences are puzzling. The modulation of additional ion channels such as TASK3 and TREK1 channels that are inhibited by zinc and functionally expressed in TC neurons may be relevant here [88,89,90].

Our findings indicate that the alternations of HCN channel properties following CPZ treatment are probably not based on the copper chelating properties of CPZ in the vicinity of HCN channels. This conclusion is in agreement with findings indicating a more complex mode of CPZ action. Administering copper as an additional supplement to the CPZ diet does not prevent formation of megamitochondria or demyelination, although copper chelation was disturbed due to CPZ feeding [43,44]. Moreover, treating primary cultures of oligodendrocytes, obtained from newborn Wistar rats, for 24–72 h with different concentrations of CPZ (maximum 1 mM), did not affect the survivability of oligodendrocytes, and further treatment of oligodendrocytes with the supernatant of astrocytes intoxicated with equivalent CPZ concentrations for 24 h also had no effect. Therefore, the presence of CPZ in an oligodendrocyte culture alone did not induce changes in cell viability [68], implying that the CPZ induced demyelination goes beyond copper deficiency.

### 4.3. Modulation of I_h_ Current by Inflammatory Cytokines-Microglial Response

One of the major hallmarks of CPZ induced demyelination is the proliferation of microglial cells. Typically, CPZ feeding induces an initial microgliosis within the first week and the number of cells continues to rise during maintained CPZ treatment up to 4 weeks [46]. The withdrawing of CPZ after 5–6 weeks rapidly reduces the number of microglia, which coincides with the peak of the remyelination phase [47]. If the CPZ diet was prolonged for 12 weeks (inducing chronic demyelination and impaired remyelination), an increased microglia number was maintained at the sites of demyelination [30]. Thus, one of the major factors of CPZ induced demyelination is a powerful microglial/macrophage response [30]. Interestingly, these cells produce tumor necrosis factor (TNF- α) and other cytokines, which partake in the pathogenesis of the CNS in demyelinating diseases. The administration of low concentrations (50 ng/mL) of TNF-α and interferon IFN-**γ,** together with 1 mM CPZ, significantly decreases the survival rate of cells in oligodendrocyte cultures. In contrast, the same cytokines without CPZ have no effect [68]. Besides detrimental effects, the immunomodulatory function of microglia and TNF-α is necessary for remyelination. In mice lacking TNF-α or its receptor (TNFR2), remyelination is significantly delayed due to the reduction of progenitor oligodendrocyte cells resulting in the reduction of mature oligodendrocytes [67]. CPZ experiments in astrocytes highlight another cytokine of the TNF superfamily. Pro-inflammatory TNF-like weak inducers of apoptosis (TWEAK) can actively induce apoptosis by binding to its receptor fibroblast growth factor-inducible 14 (Fn14). TWEAK expression is highly increased in astrocytes during CPZ treatment, and it has been proposed that TWEAK can partake in demyelination via mediating microglial accumulation [91]. Increased local IL-6 production in GFAP-IL6Tg mice reduces microglial activation during both acute and chronic demyelination, which preserves myelin. However, the impact of this preservation is questionable since most of the preserved myelin is degraded and nearly nonfunctional. Moreover, in GFAP-IL6Tg mice, CPZ treatment significantly reduced the levels of the microglial receptor referred as triggering receptor expressed on myeloid cells 2 (TREM2) and the lysosomal protein CD68. In combination with reduced microglial accumulation, this resulted in the inefficient removal of degraded myelin during remyelination and subsequently disturbed oligodendrocyte differentiation [92]. The dual role of microglia in neuroinflammation is also known for the EAE model of MS. Here, microglial IL-6 promotes immune cell infiltration and demyelination, and its ablation disrupts EAE associated inflammation [93]. Otherwise, microglial IL-4 has an anti-inflammatory role and mice with IL-4 ablation tend to be more susceptible to EAE [66].

Microglial serve different roles and release different cytokines during the demyelination and remyelination phase in the CPZ model [30]. While inflammation and demyelination are promoted in the early phase, restoration and protective processes are promoted in the late phase. Therefore, we tested cytokines typical for the two phases for their possible effect on HCN channel. While type-I interferons (IFN-α, IFN-β) and IL-1β were linked to the demyelination and remyelination phase, respectively, IL-6 is present in both phases [92,94,95,96]. Our recordings demonstrated that the investigated cytokines differentially modulate both HCN channel activity and the firing patterns of TC VB neurons. INF-α and INF-β hyperpolarized the voltage dependence of HCN channels, contributing to a significant decrease of elicited I_h_ current in native TC VB neurons (Figure 8). Moreover, the firing pattern of TC neurons is changed with INF-α by lowering the total number of action potentials and increasing the threshold for AP generation (Figure 9). Interestingly, the effects of type-I interferons on I_h_ current properties mimic the differences between currents recorded from control mice and animals on Day1 (Figure 3 and Figure 8). In comparison to INF-α and INF-β, IL-1β had opposing effects by depolarizing V_0.5_ and increasing the amplitude of I_h_ current (Figure 8). Moreover, IL-1β lowered the threshold of action potential firing in TC VB neurons and increased the total AP number in current-clamp recordings (Figure 9). Interestingly, the effects of IL-1β application on I_h_ current properties mimic the differences between currents recorded from control mice and animals on Day7 (Figure 3 and Figure 8). Intriguingly, IL-6, a cytokine with demyelinating and regenerative effects, showed no significant shifts in the properties of HCN channels (Figure 8). Therefore, our findings open up the possibility that the differences in HCN channel properties found during different phases of remyelination (Figure 3) are at least partially based on microglial responses leading to specific cytokine production. This assumption is corroborated by the finding that IFN-α and IFN-β inhibit HCN1 channels [94]. In addition, we detected an increase in Iba1-positive, activated microglia cells in the cortex of CPZ-treated C57BL/6 mice on Day1 that was decreased again on Day25 [37]. 

Nevertheless, we have not demonstrated the involvement of cytokines released by microglia in CPZ-mediated alterations of HCN channels in the present study directly. Future studies should prove the disappearance of CPZ effects on I_h_ current following genetic or pharmacological depletion of microglia [97]. Moreover, it should be noted, however, that IL-1β was found to hyperpolarize RMP, decrease the input resistance, decrease I_h_-dependent rectification, decrease the AP threshold, and decrease the number of APs in an LTS in dLGN TC neurons [49]. The reason for these diverging results is not quite clear but may be related to findings of computer simulations indicating that the decrease in amplitude of I_h_-dependent rectification is more likely to occur due to a combination of altered factors. Nevertheless, also the lower AP threshold upon IL-1β exposure was regarded as an indication of increased excitability in dLGN [49].

### 4.4. Changes of Intrathalamic Network Activity and Mathematical Modeling

In order to assess the significance of cytokine-induced changes in HCN channel properties for thalamic oscillations, we analyzed intrathalamic burst activity following stimulation of the internal capsule in horizontal slices. The application of INF-α significantly decreased the number of bursts, their duration, and the interburst frequency (Figure 10). As expected, the application IL-1β showed an opposing effect. The downregulation of intrathalamic burst activity due to INF-α can be linked to the downregulated HCN channel activity in the presence of this cytokine, especially considering that alterations of thalamocortical oscillations based on the dysregulation of HCN channels and their auxiliary subunits have been shown before [18,40]. This slowing of thalamic network oscillations is in accordance with the slowing of cortical activity in the 2–6 Hz frequency range induced by IFN-β [94]. Otherwise, the increase in I_h_ due to IL-1β application is in accordance with the increased intrathalamic burst activity (Figure 8 and Figure 10).

To prove the link between the properties of HCN channel and slow thalamic oscillations, we used a mathematical modeling approach. In a simplified model of the thalamic network, we connected two TC and two nRT neurons via AMPA and GABA_A+B_ synapses to generate slow oscillations in the delta frequency range [17,18,40,62,98]. The I_h_ current characteristics recorded during the application of inflammatory cytokines were implemented.

While qualitatively similar results were obtained for IFN-α (less oscillatory activity with the longer onset and lower frequency) and IL-1β (more oscillatory activity with shorter onset and higher frequency), no significance was reached in this regard. The reason for this difference compared to the oscillations found in horizontal slices is not completely clear. We hypothesize that the rather high variability in the combination of four different I_h_ current parameters obtained from a relatively low number of cells may contribute here. Furthermore, it is very likely that the used cytokines affect other cell properties besides I_h_ current parameters which were not incorporated into our limited 4-cell model. It is important to note that the oscillatory activity of a cell is the product of a variety of factors, including RMP, membrane resistance and a number of ionic currents. Our modeling was based only on changes in I_h_ current characteristics. According to former computational simulations, the decrease in amplitude of I_h_-dependent rectification is more likely to occur due to decreased membrane resistance in combination with decreased I_h_ conductance [49].

The increase in the number of action potentials and their frequency within a burst when modeling INF-α related parameters is rather surprising (Figure 12). LTS bursts occur mostly as a combination of the activities of T-type Ca^2+^ channels and HCN channels and even rather small modifications in these currents have a strong impact on the pattern of bursts in TC neurons [99,100]. As the T-type Ca^2+^ channels are targets of modulation by a variety of neurotransmitters and hormones, cytokines might affect them as well [49,101]. Nevertheless, our findings indicate that different proinflammatory cytokines act on TC neurons in a diverse manner including opposing modulation of HCN channels.

Our screening of a wider parameter space of I_h_ current variables points to the tendency that increasing availability of HCN channels results in more and faster intrathalamic oscillations. However, there are optimal combinations of parameters. Especially V_0.5_ shows a complex relationship with the other parameters leading to both increases and decreases in burst frequency (Figure 14). In addition, we found edge effects where the burst frequency is slightly higher near the boundary where no spontaneous oscillations occur (Figure 14). In this respect, it is interesting to note that the current density seems to influence the burst frequencies differently based on the opening kinetics (Figure 14). On the one hand, fast opening kinetics and medium current densities in the range of 10 pA/pF (as found for INF-α and Day1 (Figure 3 and Figure 8)) result in burst frequencies of 3–4 Hz. On the other hand, current densities higher than 15 pA/pF (as found during IL-1β and Day7 (Figure 3 and Figure 8)) lead to high burst frequencies of > 6 Hz (Figure 14). Importantly, the correlation between the I_h_ current density (Figure 3C) obtained from voltage-clamp recordings and the number of bursts generated by the computer model in the first second (Figure 11C) have been proved here (Figure 13). Considering this interplay of different I_h_ parameters on the outcome of burst activity, it is apparent that I_h_ current parameters have a complex interaction and modifying just one of the parameters could result in completely altered network activity.

## 5. Conclusions

CPZ induced de- and remyelination processes alter HCN channel properties in native TC VB neurons in a time dependent manner, whereas in a transient period at the early remyelination phase, HCN channels activity increased. Our experiments demonstrated that these changes are not due to the copper chelating properties of CPZ, but rather influenced by proinflammatory cytokines produced by microglial activation in response to CPZ intoxication. These cytokines also influence the firing properties of TC neurons and intrathalamic oscillations and burst activity, underlining a crucial importance of HCN channels modulation. Computational modeling, incorporating measured I_h_ properties in TC neurons, demonstrated that even a small change of I_h_ parameters could result in the altered network activity, highlighting the complex interplay between HCN channels and rhythmic thalamic bursting behavior.

## Figures and Tables

**Table 1:** The effects of divalent cation chelators on I_h_ current in TC neurons. Data are given as mean ± SEM. The control measurement and corresponding substance effect were recorded from the same cell.

		BiMPi 1 mM			EDTA 1 mM			Tricine 1 mM		
		Ctrl	BiMPi	*n*	Ctrl	EDTA	*n*	Ctrl	Tricine	*n*
V_0.5_ (mV)		−83.62 ± 0.93	−81.25 ± 1.39	8	−89.06 ± 1.49	−89.16 ± 1.33	6	−90.10 ± 1.0	−86.56 ± 0.75	7 *p* < 0.01
Current density (pA/pF)		10.86 ± 0.74	11.04 ± 0.68	8	10.54 ± 0.74	10.09 ± 0.44	6	9.50 ± 0.70	9.23 ± 0.87	7
Tau (ms)	fast	151.68 ± 7.23	145.93 ± 7.22	8	309.47 ± 20.57	316.50 ± 14.87	6	292.91 ± 15.23	276.6 ± 12.03	7
	slow	866 ± 44	886 ± 77	8	2445 ± 386	2626 ± 337	5	2407 ± 251	2130 ± 307	7

**Table 2:** The effects of zinc and phosphate-free conditions on I_h_ current in TC neurons. The data are given as mean ± SEM. Control measurement and the corresponding substance effect were recorded from the same cell.

		Zinc 0.3 mM			Phosphate-Free			
		Ctrl	Zinc	*n*	Ctrl	20 min	40 min	*n*
V_0.5_ (mV)		−87.29 ± 0.74	−84.44 ± 0.68	14 *p* < 0.001	−86.92 ± 1.30	−84.86 ± 1.10	−84.09 ± 0.95	8
Current density (pA/pF)		9.54 ± 0.36	9.63 ± 0.33	14	9.20 ± 0.54	9.85 ± 0.55	9.48 ± 0.43	8
Tau (ms)	fast	289.97 ± 13.91	285.98 ± 13.50	14	241.30 ± 14.40	234.54 ± 15.28	234.59 ± 16.84	7
	slow	2511 ± 462	1931 ± 170	13	1487 ± 167	1393 ± 122	1433 ± 124	7

**Table 3:** The effects of trace metals on I_h_ current in HEK293 cells. Data are given as mean ± SEM. The control measurement and corresponding substance effect were recorded from the same cell.

		Cu^2+^ 1 nM			Cu^2+^ 10 nM			Cu^2+^ 100 nM			Zn^2+^ 10 µM		
		Ctrl	Cu^2+^	*n*	Ctrl	Cu^2+^	*n*	Ctrl	Cu^2+^	*n*	Ctrl	Zn^2+^	*n*
V_0.5_ (mV)		−82.67 ± 2.29	−83.69 ± 1.41	5	−79.01 ± 0.38	−82.25 ± 0.68	5	−78.49 ± 0.94	−81.24 ± 1.60	8	−79.14 ± 1.75	−82.67 ± 1.98	6
Current density (pA/pF)		48.68 ± 9.61	43.59 ± 8.82	5	65.50 ± 23.20	39.58 ± 8.62	5	55.89 ± 10.54	50.13 ± 11.63	8	40.36 ± 5.82	35.22 ± 4.75	6
Tau (ms)	fast	75.37 ± 4.70	74.96 ± 7.53	5	86.06 ± 6.10	99.42 ± 8.80	5	97.16 ± 5.86	96.23 ± 10.82	8	100.18 ± 7.45	112.70 ± 7.97	6
	slow	1472 ± 224	1326 ± 120	4	1445 ± 165	1489 ± 202	3	1254 ± 193	1209 ± 93	8	1388 ± 293	1729 ± 409	4

**Table 4:** The effects of divalent cation chelators on I_h_ current in HEK293 cells. Data are given as mean ± SEM. The control measurement and corresponding substance effect were recorded from the same cell.

		BiMPi 1 mM			EDTA 1 mM		
		Ctrl	BiMPi	*n*	Ctrl	EDTA	*n*
V_0.5_ (mV)		−79.62 ± 0.74	−79.17 ± 1.39	5	−81.87 ± 1.28	−83.36 ± 1.50	11
Current density (pA/pF)		78.76 ± 13.36	72.26 ± 13.42	5	38.87 ± 5.88	33.68 ± 4.8	11
Tau (ms)	fast	112.98 ± 6.90	109.58 ± 7.31	5	96.80 ± 7.66	85.91± 6.78	11
	slow	1290 ± 201	1245 ± 117	5	1211 ± 162	952 ± 141	9

## Data Availability

The data presented in this study are available on request from the corresponding author.

## Supplementary Materials

The following supporting information can be downloaded at: https://www.mdpi.com/article/10.3390/ijms23116285/s1.

## References

[1] Browne S.H., Kang J., Akk G., Chiang L.W., Schulman H., Huguenard J.R., Prince D.A. (2001). Kinetic and pharmacological properties of GABA(A) receptors in single thalamic neurons and GABA(A) subunit expression. J. Neurophysiol..

[2] Steriade M. (1997). Synchronized activities of coupled oscillators in the cerebral cortex and thalamus at different levels of vigilance. Cereb. Cortex.

[3] Steriade M., McCormick D.A., Sejnowski T.J. (1993). Thalamocortical oscillations in the sleeping and aroused brain. Science.

[4] Llinas R.R., Ribary U., Jeanmonod D., Kronberg E., Mitra P.P. (1999). Thalamocortical dysrhythmia: A neurological and neuropsychiatric syndrome characterized by magnetoencephalography. Proc. Natl. Acad. Sci. USA.

[5] Jeanmonod D., Magnin M., Morel A. (1993). Thalamus and neurogenic pain: Physiological, anatomical and clinical data. Neuroreport.

[6] Jeanmonod D., Magnin M., Morel A. (1996). Low-threshold calcium spike bursts in the human thalamus. Common physiopathology for sensory, motor and limbic positive symptoms. Brain.

[7] Schulman J.J., Cancro R., Lowe S., Lu F., Walton K.D., Llinas R.R. (2011). Imaging of thalamocortical dysrhythmia in neuropsychiatry. Front. Hum. Neurosci..

[8] Broicher T., Seidenbecher T., Meuth P., Munsch T., Meuth S.G., Kanyshkova T., Pape H.C., Budde T. (2007). T-current related effects of antiepileptic drugs and a Ca^2+^ channel antagonist on thalamic relay and local circuit interneurons in a rat model of absence epilepsy. Neuropharmacology.

[9] Kanyshkova T., Meuth P., Bista P., Liu Z., Ehling P., Caputi L., Doengi M., Chetkovich D.M., Pape H.C., Budde T. (2012). Differential regulation of HCN channel isoform expression in thalamic neurons of epileptic and non-epileptic rat strains. Neurobiol. Dis..

[10] Perez-Reyes E. (2003). Molecular physiology of low-voltage-activated t-type calcium channels. Physiol. Rev..

[11] Lory P., Nicole S., Monteil A. (2020). Neuronal Cav3 channelopathies: Recent progress and perspectives. Pflugers Arch..

[12] Spinelli V., Sartiani L., Mugelli A., Romanelli M.N., Cerbai E. (2018). Hyperpolarization-activated cyclic-nucleotide-gated channels: Pathophysiological, developmental, and pharmacological insights into their function in cellular excitability. Can. J. Physiol. Pharmacol..

[13] Sartiani L., Mannaioni G., Masi A., Novella Romanelli M., Cerbai E. (2017). The Hyperpolarization-Activated Cyclic Nucleotide-Gated Channels: From Biophysics to Pharmacology of a Unique Family of Ion Channels. Pharmacol. Rev..

[14] Llinas R.R., Steriade M. (2006). Bursting of thalamic neurons and states of vigilance. J. Neurophysiol..

[15] Ludwig A., Budde T., Stieber J., Moosmang S., Wahl C., Holthoff K., Langebartels A., Wotjak C., Munsch T., Zong X. (2003). Absence epilepsy and sinus dysrhythmia in mice lacking the pacemaker channel HCN_2_. EMBO J..

[16] Heuermann R.J., Jaramillo T.C., Ying S.W., Suter B.A., Lyman K.A., Han Y., Lewis A.S., Hampton T.G., Shepherd G.M.G., Goldstein P.A. (2016). Reduction of thalamic and cortical Ih by deletion of TRIP8b produces a mouse model of human absence epilepsy. Neurobiol. Dis..

[17] Zobeiri M., Chaudhary R., Blaich A., Rottmann M., Herrmann S., Meuth P., Bista P., Kanyshkova T., Luttjohann A., Narayanan V. (2019). The Hyperpolarization-Activated HCN4 Channel is Important for Proper Maintenance of Oscillatory Activity in the Thalamocortical System. Cereb. Cortex.

[18] Zobeiri M., Chaudhary R., Datunashvili M., Heuermann R.J., Luttjohann A., Narayanan V., Balfanz S., Meuth P., Chetkovich D.M., Pape H.C. (2018). Modulation of thalamocortical oscillations by TRIP8b, an auxiliary subunit for HCN channels. Brain Struct. Funct..

[19] Datunashvili M., Chaudhary R., Zobeiri M., Luttjohann A., Mergia E., Baumann A., Balfanz S., Budde B., van Luijtelaar G., Pape H.C. (2018). Modulation of Hyperpolarization-Activated Inward Current and Thalamic Activity Modes by Different Cyclic Nucleotides. Front. Cell. Neurosci..

[20] De Ridder D., Vanneste S., Langguth B., Llinas R. (2015). Thalamocortical Dysrhythmia: A Theoretical Update in Tinnitus. Front. Neurol..

[21] Zhang D., Choi Y.S., Madhok J., Jia X., Koenig M., Thakor N. (2009). Neural signals in cortex and thalamus during brain injury from cardiac arrest in rats. Annu. Int. Conf. IEEE Eng. Med. Biol. Soc..

[22] Chung W.K., Shin M., Jaramillo T.C., Leibel R.L., LeDuc C.A., Fischer S.G., Tzilianos E., Gheith A.A., Lewis A.S., Chetkovich D.M. (2009). Absence epilepsy in apathetic, a spontaneous mutant mouse lacking the h channel subunit, HCN_2_. Neurobiol. Dis..

[23] Barnett M.H., Prineas J.W. (2004). Relapsing and remitting multiple sclerosis: Pathology of the newly forming lesion. Ann. Neurol..

[24] Kipp M., Wagenknecht N., Beyer C., Samer S., Wuerfel J., Nikoubashman O. (2015). Thalamus pathology in multiple sclerosis: From biology to clinical application. Cell. Mol. Life Sci..

[25] Chard D.T., Alahmadi A.A.S., Audoin B., Charalambous T., Enzinger C., Hulst H.E., Rocca M.A., Rovira A., Sastre-Garriga J., Schoonheim M.M. (2021). Mind the gap: From neurons to networks to outcomes in multiple sclerosis. Nat. Rev. Neurol..

[26] Deppe M., Kramer J., Tenberge J.G., Marinell J., Schwindt W., Deppe K., Groppa S., Wiendl H., Meuth S.G. (2016). Early silent microstructural degeneration and atrophy of the thalamocortical network in multiple sclerosis. Hum. Brain Mapp..

[27] Capone F., Collorone S., Cortese R., Di Lazzaro V., Moccia M. (2020). Fatigue in multiple sclerosis: The role of thalamus. Mult. Scler..

[28] Roostaei T., Sadaghiani S., Park M.T., Mashhadi R., Nazeri A., Noshad S., Salehi M.J., Naghibzadeh M., Moghadasi A.N., Owji M. (2016). Channelopathy-related SCN10A gene variants predict cerebellar dysfunction in multiple sclerosis. Neurology.

[29] Correale J., Marrodan M., Benarroch E.E. (2020). What is the role of axonal ion channels in multiple sclerosis?. Neurology.

[30] Plastini M.J., Desu H.L., Brambilla R. (2020). Dynamic Responses of Microglia in Animal Models of Multiple Sclerosis. Front. Cell. Neurosci..

[31] Kipp M. (2016). Remyelination strategies in multiple sclerosis: A critical reflection. Expert Rev. Neurother..

[32] Hamada M.S., Goethals S., de Vries S.I., Brette R., Kole M.H. (2016). Covariation of axon initial segment location and dendritic tree normalizes the somatic action potential. Proc. Natl. Acad. Sci. USA.

[33] Bagchi B., Al-Sabi A., Kaza S., Scholz D., O’Leary V.B., Dolly J.O., Ovsepian S.V. (2014). Disruption of myelin leads to ectopic expression of K(V)1.1 channels with abnormal conductivity of optic nerve axons in a cuprizone-induced model of demyelination. PLoS ONE.

[34] Tourdias T., Mori N., Dragonu I., Cassagno N., Boiziau C., Aussudre J., Brochet B., Moonen C., Petry K.G., Dousset V. (2011). Differential aquaporin 4 expression during edema build-up and resolution phases of brain inflammation. J. Neuroinflamm..

[35] Ellwardt E., Pramanik G., Luchtman D., Novkovic T., Jubal E.R., Vogt J., Arnoux I., Vogelaar C.F., Mandal S., Schmalz M. (2018). Maladaptive cortical hyperactivity upon recovery from experimental autoimmune encephalomyelitis. Nat. Neurosci..

[36] Ghaffarian N., Mesgari M., Cerina M., Gobel K., Budde T., Speckmann E.J., Meuth S.G., Gorji A. (2016). Thalamocortical-auditory network alterations following cuprizone-induced demyelination. J. Neuroinflamm..

[37] Cerina M., Narayanan V., Gobel K., Bittner S., Ruck T., Meuth P., Herrmann A.M., Stangel M., Gudi V., Skripuletz T. (2017). The quality of cortical network function recovery depends on localization and degree of axonal demyelination. Brain Behav. Immun..

[38] Narayanan V., Cerina M., Gobel K., Meuth P., Herrmann A.M., Fernandez-Orth J., Stangel M., Gudi V., Skripuletz T., Daldrup T. (2018). Impairment of frequency-specific responses associated with altered electrical activity patterns in auditory thalamus following focal and general demyelination. Exp. Neurol..

[39] Cerina M., Narayanan V., Delank A., Meuth P., Graebenitz S., Gobel K., Herrmann A.M., Albrecht S., Daldrup T., Seidenbecher T. (2018). Protective potential of dimethyl fumarate in a mouse model of thalamocortical demyelination. Brain Struct. Funct..

[40] Chaudhary R., Albrecht S., Datunashvili M., Cerina M., Luttjohann A., Han Y., Narayanan V., Chetkovich D.M., Ruck T., Kuhlmann T. (2022). Modulation of Pacemaker Channel Function in a Model of Thalamocortical Hyperexcitability by Demyelination and Cytokines. Cereb. Cortex.

[41] Praet J., Guglielmetti C., Berneman Z., Van der Linden A., Ponsaerts P. (2014). Cellular and molecular neuropathology of the cuprizone mouse model: Clinical relevance for multiple sclerosis. Neurosci. Biobehav. Rev..

[42] Vega-Riquer J.M., Mendez-Victoriano G., Morales-Luckie R.A., Gonzalez-Perez O. (2019). Five Decades of Cuprizone, an Updated Model to Replicate Demyelinating Diseases. Curr. Neuropharmacol..

[43] Carlton W.W. (1967). Studies on the induction of hydrocephalus and spongy degeneration by cuprizone feeding and attempts to antidote the toxicity. Life Sci..

[44] Carey E.M., Freeman N.M. (1983). Biochemical changes in Cuprizone-induced spongiform encephalopathy. I. Changes in the activities of 2’,3’-cyclic nucleotide 3’-phosphohydrolase, oligodendroglial ceramide galactosyl transferase, and the hydrolysis of the alkenyl group of alkenyl, acyl-glycerophospholipids by plasmalogenase in different regions of the brain. Neurochem. Res..

[45] Zatta P., Raso M., Zambenedetti P., Wittkowski W., Messori L., Piccioli F., Mauri P.L., Beltramini M. (2005). Copper and zinc dismetabolism in the mouse brain upon chronic cuprizone treatment. Cell. Mol. Life Sci..

[46] Hiremath M.M., Saito Y., Knapp G.W., Ting J.P., Suzuki K., Matsushima G.K. (1998). Microglial/macrophage accumulation during cuprizone-induced demyelination in C57BL/6 mice. J. Neuroimmunol..

[47] Mason J.L., Jones J.J., Taniike M., Morell P., Suzuki K., Matsushima G.K. (2000). Mature oligodendrocyte apoptosis precedes IGF-1 production and oligodendrocyte progenitor accumulation and differentiation during demyelination/remyelination. J. Neurosci. Res..

[48] Tezuka T., Tamura M., Kondo M.A., Sakaue M., Okada K., Takemoto K., Fukunari A., Miwa K., Ohzeki H., Kano S. (2013). Cuprizone short-term exposure: Astrocytic IL-6 activation and behavioral changes relevant to psychosis. Neurobiol. Dis..

[49] Samios V.N., Inoue T. (2014). Interleukin-1beta and interleukin-6 affect electrophysiological properties of thalamic relay cells. Neurosci. Res..

[50] Vemana S., Pandey S., Larsson H.P. (2008). Intracellular Mg^2+^ is a voltage-dependent pore blocker of HCN channels. Am. J. Physiol. Cell Physiol..

[51] Notomi T., Kuno M., Hiyama A., Ohura K., Noda M., Skerry T.M. (2015). Zinc-Induced Effects on Osteoclastogenesis Involves Activation of Hyperpolarization-Activated Cyclic Nucleotide Modulated Channels via Changes in Membrane Potential. J. Bone Miner. Res..

[52] Notomi T., Hiyama A., Nozaki T. (2019). Role of zinc and zinc-modulated ion channels, ORAI1 and HCN in osteoclasts. J. Transl. Sci..

[53] Seifert R., Scholten A., Gauss R., Mincheva A., Lichter P., Kaupp U.B. (1999). Molecular characterization of a slowly gating human hyperpolarization-activated channel predominantly expressed in thalamus, heart, and testis. Proc. Natl. Acad. Sci. USA.

[54] Fries M., Mertens M., Teske N., Kipp M., Beyer C., Willms T., Valkonen A., Rissanen K., Albrecht M., Clarner T. (2019). Water-Soluble Cuprizone Derivative: Synthesis, Characterization, and in Vitro Studies. ACS Omega.

[55] Neumaier F., Akhtar-Schafer I., Luke J.N., Dibue-Adjei M., Hescheler J., Schneider T. (2018). Reciprocal modulation of Cav 2.3 voltage-gated calcium channels by copper(II) ions and kainic acid. J. Neurochem..

[56] Hammelmann V., Stieglitz M.S., Hulle H., Le Meur K., Kass J., Brummer M., Gruner C., Rotzer R.D., Fenske S., Hartmann J. (2019). Abolishing cAMP sensitivity in HCN_2_ pacemaker channels induces generalized seizures. JCI Insight.

[57] Fenske S., Hennis K., Rotzer R.D., Brox V.F., Becirovic E., Scharr A., Gruner C., Ziegler T., Mehlfeld V., Brennan J. (2020). cAMP-dependent regulation of HCN4 controls the tonic entrainment process in sinoatrial node pacemaker cells. Nat. Commun..

[58] Moller M., Silbernagel N., Wrobel E., Stallmayer B., Amedonu E., Rinne S., Peischard S., Meuth S.G., Wunsch B., Strutz-Seebohm N. (2018). In Vitro Analyses of Novel HCN_4_ Gene Mutations. Cell. Physiol. Biochem..

[59] Neumaier F., Alpdogan S., Hescheler J., Schneider T. (2018). A practical guide to the preparation and use of metal ion-buffered systems for physiological research. Acta Physiol..

[60] Rumschik S.M., Nydegger I., Zhao J., Kay A.R. (2009). The interplay between inorganic phosphate and amino acids determines zinc solubility in brain slices. J. Neurochem..

[61] Hines M.L., Carnevale N.T. (2001). NEURON: A tool for neuroscientists. Neuroscientist.

[62] Destexhe A., Bal T., McCormick D.A., Sejnowski T.J. (1996). Ionic mechanisms underlying synchronized oscillations and propagating waves in a model of ferret thalamic slices. J. Neurophysiol..

[63] Skripuletz T., Gudi V., Hackstette D., Stangel M. (2011). De- and remyelination in the CNS white and grey matter induced by cuprizone: The old, the new, and the unexpected. Histol. Histopathol..

[64] Gibbs J.W., Zhang Y.F., Shumate M.D., Coulter D.A. (2000). Regionally selective blockade of GABAergic inhibition by zinc in the thalamocortical system: Functional significance. J. Neurophysiol..

[65] Meuth S.G., Aller M.I., Munsch T., Schuhmacher T., Seidenbecher T., Meuth P., Kleinschnitz C., Pape H.C., Wiendl H., Wisden W. (2006). The contribution of TWIK-related acid-sensitive K^+^-containing channels to the function of dorsal lateral geniculate thalamocortical relay neurons. Mol. Pharmacol..

[66] Ponomarev E.D., Maresz K., Tan Y., Dittel B.N. (2007). CNS-derived interleukin-4 is essential for the regulation of autoimmune inflammation and induces a state of alternative activation in microglial cells. J. Neurosci..

[67] Arnett H.A., Mason J., Marino M., Suzuki K., Matsushima G.K., Ting J.P. (2001). TNF alpha promotes proliferation of oligodendrocyte progenitors and remyelination. Nat. Neurosci..

[68] Pasquini L.A., Calatayud C.A., Bertone Una A.L., Millet V., Pasquini J.M., Soto E.F. (2007). The neurotoxic effect of cuprizone on oligodendrocytes depends on the presence of pro-inflammatory cytokines secreted by microglia. Neurochem. Res..

[69] Byczkowicz N., Eshra A., Montanaro J., Trevisiol A., Hirrlinger J., Kole M.H., Shigemoto R., Hallermann S. (2019). HCN channel-mediated neuromodulation can control action potential velocity and fidelity in central axons. Elife.

[70] Frigerio F., Flynn C., Han Y., Lyman K., Lugo J.N., Ravizza T., Ghestem A., Pitsch J., Becker A., Anderson A.E. (2018). Neuroinflammation Alters Integrative Properties of Rat Hippocampal Pyramidal Cells. Mol. Neurobiol..

[71] Doucette J.R., Jiao R., Nazarali A.J. (2010). Age-related and cuprizone-induced changes in myelin and transcription factor gene expression and in oligodendrocyte cell densities in the rostral corpus callosum of mice. Cell. Mol. Neurobiol..

[72] Wang H., Li C., Wang H., Mei F., Liu Z., Shen H.Y., Xiao L. (2013). Cuprizone-induced demyelination in mice: Age-related vulnerability and exploratory behavior deficit. Neurosci. Bull..

[73] Ludwin S.K. (1978). Central nervous system demyelination and remyelination in the mouse: An ultrastructural study of cuprizone toxicity. Lab. Investig..

[74] Taylor L.C., Gilmore W., Matsushima G.K. (2009). SJL mice exposed to cuprizone intoxication reveal strain and gender pattern differences in demyelination. Brain Pathol..

[75] Skripuletz T., Lindner M., Kotsiari A., Garde N., Fokuhl J., Linsmeier F., Trebst C., Stangel M. (2008). Cortical demyelination is prominent in the murine cuprizone model and is strain-dependent. Am. J. Pathol..

[76] Taylor L.C., Gilmore W., Ting J.P., Matsushima G.K. (2010). Cuprizone induces similar demyelination in male and female C57BL/6 mice and results in disruption of the estrous cycle. J. Neurosci. Res..

[77] Santoro B., Wainger B.J., Siegelbaum S.A. (2004). Regulation of HCN channel surface expression by a novel C-terminal protein-protein interaction. J. Neurosci..

[78] Santoro B., Piskorowski R.A., Pian P., Hu L., Liu H., Siegelbaum S.A. (2009). TRIP8b splice variants form a family of auxiliary subunits that regulate gating and trafficking of HCN channels in the brain. Neuron.

[79] Foote K.M., Lyman K.A., Han Y., Michailidis I.E., Heuermann R.J., Mandikian D., Trimmer J.S., Swanson G.T., Chetkovich D.M. (2019). Phosphorylation of the HCN channel auxiliary subunit TRIP8b is altered in an animal model of temporal lobe epilepsy and modulates channel function. J. Biol. Chem..

[80] Mathie A., Sutton G.L., Clarke C.E., Veale E.L. (2006). Zinc and copper: Pharmacological probes and endogenous modulators of neuronal excitability. Pharmacol. Ther..

[81] Traboulsie A., Chemin J., Chevalier M., Quignard J.F., Nargeot J., Lory P. (2007). Subunit-specific modulation of T-type calcium channels by zinc. J. Physiol..

[82] Castelli L., Tanzi F., Taglietti V., Magistretti J. (2003). Cu^2+^, Co^2+^, and Mn^2+^ modify the gating kinetics of high-voltage-activated Ca^2+^ channels in rat palaeocortical neurons. J. Membr. Biol..

[83] Neumaier F., Dibue-Adjei M., Hescheler J., Schneider T. (2015). Voltage-gated calcium channels: Determinants of channel function and modulation by inorganic cations. Prog. Neurobiol..

[84] Neumaier F., Schneider T., Albanna W. (2020). Cav2.3 channel function and Zn(^2+^)-induced modulation: Potential mechanisms and (patho)physiological relevance. Channels.

[85] Cataldi M., Lariccia V., Marzaioli V., Cavaccini A., Curia G., Viggiano D., Canzoniero L.M., di Renzo G., Avoli M., Annunziato L. (2007). Zn(^2+^) slows down Ca(V)3.3 gating kinetics: Implications for thalamocortical activity. J. Neurophysiol..

[86] Gibbs J.W., Schroder G.B., Coulter D.A. (1996). GABAA receptor function in developing rat thalamic reticular neurons: Whole cell recordings of GABA-mediated currents and modulation by clonazepam. J. Neurophysiol..

[87] Zucconi G.G., Cipriani S., Scattoni R., Balgkouranidou I., Hawkins D.P., Ragnarsdottir K.V. (2007). Copper deficiency elicits glial and neuronal response typical of neurodegenerative disorders. Neuropathol. Appl. Neurobiol..

[88] Bista P., Pawlowski M., Cerina M., Ehling P., Leist M., Meuth P., Aissaoui A., Borsotto M., Heurteaux C., Decher N. (2015). Differential phospholipase C-dependent modulation of TASK and TREK two-pore domain K^+^ channels in rat thalamocortical relay neurons. J. Physiol..

[89] Musset B., Meuth S.G., Liu G.X., Derst C., Wegner S., Pape H.C., Budde T., Preisig-Muller R., Daut J. (2006). Effects of divalent cations and spermine on the K^+^ channel TASK-3 and on the outward current in thalamic neurons. J. Physiol..

[90] Gruss M., Mathie A., Lieb W.R., Franks N.P. (2004). The two-pore-domain K(^+^) channels TREK-1 and TASK-3 are differentially modulated by copper and zinc. Mol. Pharmacol..

[91] Iocca H.A., Plant S.R., Wang Y., Runkel L., O’Connor B.P., Lundsmith E.T., Hahm K., van Deventer H.W., Burkly L.C., Ting J.P. (2008). TNF superfamily member TWEAK exacerbates inflammation and demyelination in the cuprizone-induced model. J. Neuroimmunol..

[92] Petkovic F., Campbell I.L., Gonzalez B., Castellano B. (2016). Astrocyte-targeted production of interleukin-6 reduces astroglial and microglial activation in the cuprizone demyelination model: Implications for myelin clearance and oligodendrocyte maturation. Glia.

[93] Sanchis P., Fernandez-Gayol O., Comes G., Escrig A., Giralt M., Palmiter R.D., Hidalgo J. (2020). Interleukin-6 Derived from the Central Nervous System May Influence the Pathogenesis of Experimental Autoimmune Encephalomyelitis in a Cell-Dependent Manner. Cells.

[94] Stadler K., Bierwirth C., Stoenica L., Battefeld A., Reetz O., Mix E., Schuchmann S., Velmans T., Rosenberger K., Brauer A.U. (2014). Elevation in type I interferons inhibits HCN1 and slows cortical neuronal oscillations. Cereb. Cortex.

[95] Vezzani A., Maroso M., Balosso S., Sanchez M.A., Bartfai T. (2011). IL-1 receptor/Toll-like receptor signaling in infection, inflammation, stress and neurodegeneration couples hyperexcitability and seizures. Brain Behav. Immun..

[96] Mason J.L., Suzuki K., Chaplin D.D., Matsushima G.K. (2001). Interleukin-1beta promotes repair of the CNS. J. Neurosci..

[97] Li S., Liao Y., Dong Y., Li X., Li J., Cheng Y., Cheng J., Yuan Z. (2021). Microglial deletion and inhibition alleviate behavior of post-traumatic stress disorder in mice. J. Neuroinflamm..

[98] Destexhe A., Contreras D., Steriade M. (1998). Mechanisms underlying the synchronizing action of corticothalamic feedback through inhibition of thalamic relay cells. J. Neurophysiol..

[99] Tscherter A., David F., Ivanova T., Deleuze C., Renger J.J., Uebele V.N., Shin H.S., Bal T., Leresche N., Lambert R.C. (2011). Minimal alterations in T-type calcium channel gating markedly modify physiological firing dynamics. J. Physiol..

[100] Choi S., Yu E., Lee S., Llinas R.R. (2015). Altered thalamocortical rhythmicity and connectivity in mice lacking CaV3.1 T-type Ca^2+^ channels in unconsciousness. Proc. Natl. Acad. Sci. USA.

[101] Errington A.C., Hughes S.W., Crunelli V. (2012). Rhythmic dendritic Ca^2+^ oscillations in thalamocortical neurons during slow non-REM sleep-related activity in vitro. J. Physiol..

