# Supplementary material for: Effects of Axonal Demyelination, Inflammatory Cytokines and Divalent Cation Chelators on Thalamic HCN Channels and Oscillatory Bursting
Source: Int J Mol Sci. 2022 Jun 3;23(11):6285. doi: 10.3390/ijms23116285 (PMC9181513; doi:10.3390/ijms23116285)
Supplement: Supplementary file 1 [file ijms-23-06285-s001.zip › ijms-1724156-supplementary.pdf]

SUPPLEMENTARY DATA

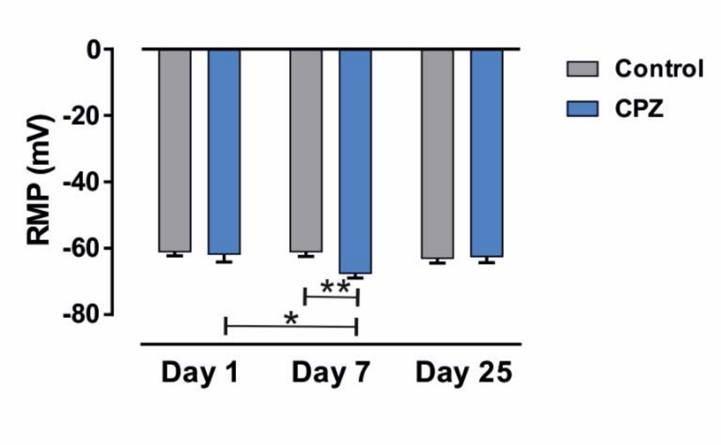

Supplementary Figure S1: Impact of CPZ-induced de- and remyelination on the resting membrane potential (RMP) of the thalamic neurons. *Bar graphs displaying the resting membrane potential for CPZ - treated mice (blue) after 1, 7, 25 days of remyelination and their aged matched controls (grey) for each remyelination period.*

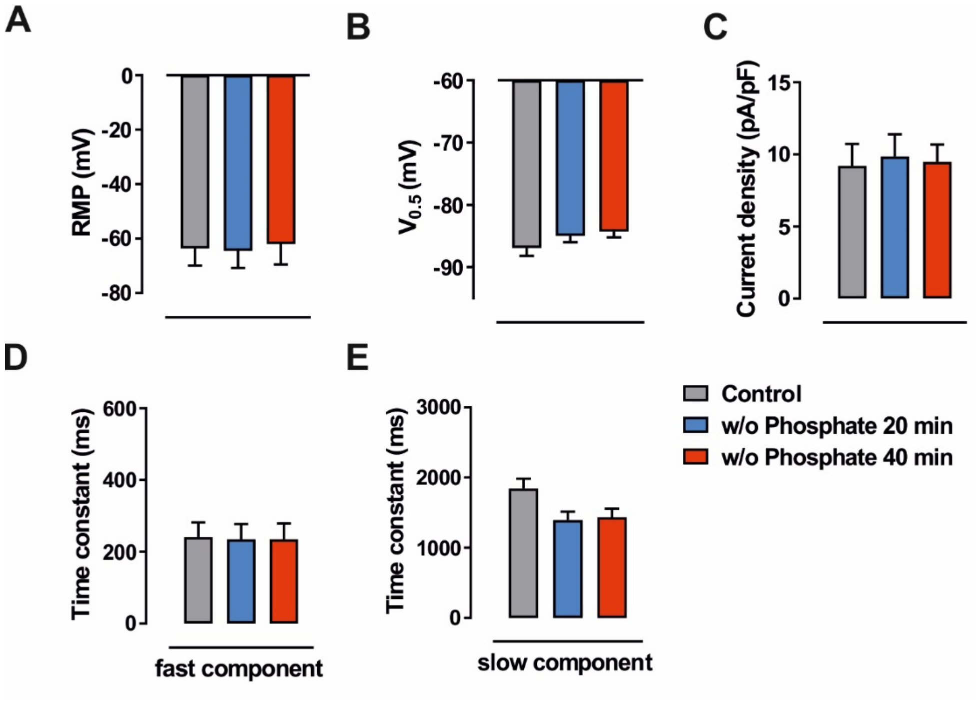

Supplementary Figure S2: Impact of nominally phosphate-free ACSF on I_h current recorded from VB TC neurons. *(A-E) Bar graphs showing the resting membrane potential (A), $V_{0.5}$ (B), current density (C), fast (D) and slow (E) activation kinetics of I_h current recorded from VB TC neurons under control condition (grey), after 20 min (blue) and 40 min (red) wash-in of nominally phosphate-free ACSF.*

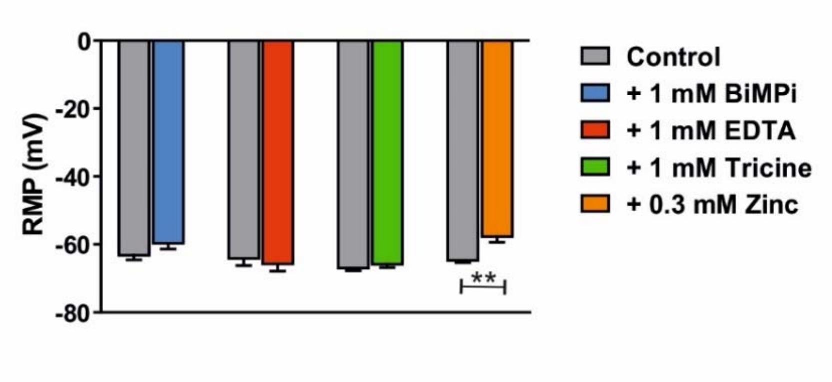

Supplementary Figure S3: Effects of divalent cation chelators and trace metals on the resting membrane potential (RMP) of the thalamic neurons. *Bar graphs comparing the RMP in control and experimental conditions (as indicated).*

Supplementary Table S1: Effects of CPZ on I_h current recorded in oocytes expressing hHCN4 channels*. Mean current amplitudes [nA ± SEM] in presence of ND96 + 0.2 % DMSO (ctrl) and 100 µM CPZ (+CPZ). Mean amplitudes were derived from n independent oocytes.*

		Mean current amplitudes [nA] ± SEM					
	n	-40 mV	-60 mV	-80 mV	-100 mV	-120 mV	-140 mV
Ctrl	11	18 ± 5	-25 ± 7	-95 ± 14	-256 ± 29	-522 ± 63	-907 ± 116
+CPZ	8	20 ± 11	-26 ± 17	-94 ± 25	-272 ± 43	-576 ± 89	-957 ± 159
